# An Energy-Efficient Spectrum-Aware Reinforcement Learning-Based Clustering Algorithm for Cognitive Radio Sensor Networks

**DOI:** 10.3390/s150819783

**Published:** 2015-08-13

**Authors:** Ibrahim Mustapha, Borhanuddin Mohd Ali, Mohd Fadlee A. Rasid, Aduwati Sali, Hafizal Mohamad

**Affiliations:** 1 Department of Computer and Communications Systems Engineering and Wireless and Photonics Research Centre, Faculty of Engineering, Universiti Putra Malaysia, 43400 Serdang Selangor, Malaysia; E-Mails: mustib@unimaid.edu.ng (I.M.); fadlee@upm.edu.my (M.F.A.R.); aduwati@upm.edu.my (A.S.); 2 Department of Electrical and Electronics Engineering, Faculty of Engineering, University of Maiduguri, P. M. B. 1069, Maiduguri, Nigeria; 3 Wireless Networks and Protocol Research Lab, MIMOS Berhad, Technology Park Malaysia, 57000 Kuala Lumpur, Malaysia; E-Mail: hafizal.mohamad@mimos.my

**Keywords:** clustering, reinforcement learning, energy consumption, cooperative sensing, wireless sensor network, cognitive radio

## Abstract

It is well-known that clustering partitions network into logical groups of nodes in order to achieve energy efficiency and to enhance dynamic channel access in cognitive radio through cooperative sensing. While the topic of energy efficiency has been well investigated in conventional wireless sensor networks, the latter has not been extensively explored. In this paper, we propose a reinforcement learning-based spectrum-aware clustering algorithm that allows a member node to learn the energy and cooperative sensing costs for neighboring clusters to achieve an optimal solution. Each member node selects an optimal cluster that satisfies pairwise constraints, minimizes network energy consumption and enhances channel sensing performance through an exploration technique. We first model the network energy consumption and then determine the optimal number of clusters for the network. The problem of selecting an optimal cluster is formulated as a Markov Decision Process (MDP) in the algorithm and the obtained simulation results show convergence, learning and adaptability of the algorithm to dynamic environment towards achieving an optimal solution. Performance comparisons of our algorithm with the Groupwise Spectrum Aware (GWSA)-based algorithm in terms of Sum of Square Error (SSE), complexity, network energy consumption and probability of detection indicate improved performance from the proposed approach. The results further reveal that an energy savings of 9% and a significant Primary User (PU) detection improvement can be achieved with the proposed approach.

## 1. Introduction

Technological advances in microelectronics have led to the widespread applications of wireless sensor networks (WSNs) in a variety of application areas. In general, wireless sensor nodes and many other wireless devices based on Wi-Fi, Zigbee and Bluetooth standards operate in unlicensed spectrum bands such as the Industrial Scientific and Medical (ISM) band which lack tight regulations. This leads to severe congestion in the useable unlicensed spectrum bands and causes harmful interference between the various wireless devices. On the other hand, licensed spectrum bands which are assigned to licensed users known as Primary Users (PUs) tend to become underutilized due to their fixed spectrum band allocation, as reported in the Federal Communications Commission (FCC) report [1]. This necessitates the need for a paradigm shift from the conventional inefficient spectrum allocation policy to a dynamic and more flexible spectrum access management.

Cognitive Radio (CR) is a new paradigm that has the potential to efficiently utilize the unused licensed spectrum bands, also known as spectrum holes, by dynamically allocating the spectrum holes to unlicensed users referred to as Secondary Users (SUs) without any harmful interference with PUs’ transmissions. Therefore, the main motivation for CR is dynamic access to temporal and spatial spectrum holes [2]. Spectrum sensing is the main fundamental function of CR for spectrum band exploration to identify spectrum holes and to protect PUs from harmful interference. Two performance metrics namely, probability of detection *P_d_* and probability of false alarm *P_f_* are used to measure the reliability of spectrum sensing techniques for discovering the availability or otherwise of spectrum holes. However, wireless propagation impairments such as multi-path fading, receiver uncertainty, shadowing and interference in wireless channels degrade the performance of PU detection techniques [3].

Cooperative spectrum sensing is a promising approach to overcome these problems [4]. The approach involves coordinating multiple CRs to share their local sensing results and make a collective decision about spectrum hole availability. It also improves the probability of PU detection through exploration of multi-users’ sensing diversity. A Fusion Centre (FC) performs decision fusion on the sensing results obtained and makes a global decision on the spectrum holes’ status. Although cooperative spectrum sensing yields better sensing performance, it also increases communications overhead, incurs in high energy consumption as well as extra sensing and reporting delays, particularly in large-scale networks such as CR-WSN. These problems can be minimized by logical grouping of multiple SUs to form a cluster. 

CR-WSN is a network of dispersed wireless sensor nodes embedded with cognitive radio capability which enable them to dynamically access unused licensed spectrum bands for data transmission while performing conventional wireless sensor nodes’ tasks [5]. CR-WSN offers several potential benefits to a wide range of applications domains and has been proposed as one of the most promising technologies to address spectrum access and utilization challenges in WSN [6,7]. For instance, when multiple conventional sensor nodes attempt to simultaneously transmit data through the overcrowded unlicensed spectrum bands, the transmitted packets may not get to the destination due to packet collisions. This not only leads to excessive network power consumption as a result of packet retransmissions, but also increases the probability of packet collisions which significantly affect the communication reliability of the network [8]. Although, cognitive radio sensor nodes can dynamically access multiple unused licensed channels for data transmission in order to mitigate this challenge, the additional task of opportunistic access to unused licensed channels through spectrum sensing incurs a significant energy cost. This means that CR-WSN inevitably consumes much more energy than conventional WSN due to the cognitive capability. 

Generally, cognitive radio sensor nodes are characterized by limited energy, constrained storage and processing resources, which are inherited from conventional wireless sensor nodes. Therefore, the main challenges in CR-WSNs are energy efficient communications to extend the lifetime of the network and PU protection from unlawful interference.

Network clustering involves partitioning the network into logical groups of nodes that form clusters, each cluster comprises of a clusterhead (CH) while the none clusterhead nodes are referred to as Member Nodes (MNs). The CH may serve as a central point to all nodes in the cluster, and it performs various tasks such as data aggregation and spectrum sensing coordination. In addition, it also provides inter-cluster communications by communicating with neighboring CHs and a Base Station (BS). The MN detects events and communicates its data to the associated CH through intra-cluster communications through either single-hop or multi-hop routing. 

The network clustering process generally involves three phases: initialization, setup and maintenance phases, which gives the main distinction among the various clustering algorithms. The initialization phase can either be centralized or distributed. The setup phase involves emergence of CHs based on either pre-defined metric functions or random selection, it also involves formation of MNs in the cluster where each MN joins its respective cluster either by default or based on some metric function. Finally, the maintenance phase deals with rotation of nodes’ roles and re-clustering of the network when a pre-defined condition is reached or at the beginning of every round. 

Clustering of a network has several benefits and it has been widely explored in conventional wireless sensor networks; they are firstly to achieve network scalability [9], and at the same time prolong the lifetime of a network [8]. However, its application in CR-WSN to enhance PU protection has not been fully explored [10]. Existing clustering algorithms mainly focus on routing [11] and energy consumption issues in conventional WSNs [12], and only a few have attempted to address both energy consumption and spectrum sensing performance issues in CR-WSN. 

Therefore, conventional clustering algorithms for WSNs or mobile *ad hoc* networks may not be suitable for CR-WSN due to the dynamic nature of the channels. This necessitates the need for a novel clustering algorithm that will address both energy issues and spectrum holes detection issues in CR-WSN. Network clustering to support many cognitive radio tasks such as dynamic channels access, cooperative sensing and routing has been extensively discussed in [13]. Cognitive radio-based network clustering requires additional conditions for grouping of nodes based on common vacant channels detected in a temporal and spatial neighborhood [14]. In such a network, sensor nodes within a cluster are require to have at least one common vacant channel between the transmitter and the receiver for communication. The dynamic nature of the environment which is influenced by the PU activities necessitates the need for spectrum aware clustering schemes. 

Spectrum-aware clustering schemes in cognitive radio networks has received considerable attention in recent times. Network scalability and heterogeneity challenges have been well investigated and addressed such as in [15]. The scheme is based on distributed coordination approach where SUs construct groups in accordance with common vacant channels locally detected. A spectrum-aware routing solution for cognitive radio described in [16] selects routes that offer the highest spectrum availability and computes its long-term routing metrics to balance between short-term route performance and long term route satiability. The scheme mainly addresses channelization and dynamic variation issues in cognitive radio routing protocol to effectively utilize unused licensed channels. The spectrum aware clustering scheme in [14] is mainly driven by an event which requires a temporal cluster. The scheme uses nodes’ local position in respect to the event and sinks to select eligible nodes for clustering. It then elects a clusterhead among the eligible nodes based on channel availability, node degree and distance to sinks in the neighborhood. The authors in [17] proposed a centralized Groupwise Spectrum-Aware (GWSA) clustering algorithm; it first creates a proximity matrix for all nodes, determines from the matrix the global minimum distance between pairs and then merges the nearest clusters that satisfy the Groupwise constraints in each iteration until an optimal number of clusters that minimizes network-wide energy consumption is achieved. 

However, this approach suffers from network instability because PU arrival may cause re-clustering of the whole network and also its high computational complexity which increases proportionately along with the size of the proximity matrix limits its practical implementation in a large-scale network. The algorithm described in [18] minimizes the network instability problem such that re-clustering involves only nodes that detect the PU arrival while network topology of other nodes remains intact. Furthermore, it determines local minimum distance between neighboring cluster pair and merges multiple nearest cluster pair at a single time to increase the convergence rate. Although the algorithm achieves relatively much less computation complexity, its performance is relatively inferior to the GWSA. Unlike [17,18] our proposed algorithm not only tends to minimize network energy consumption, but also improves spectrum hole detection, by way of exploring multi-user sensing diversity through cooperative spectrum sensing which is essential for dynamic spectrum access in cognitive radio.

The reinforcement learning (RL) technique has been applied to many existing works related to cognitive radio, but they were mainly applied to spectrum sensing [2], cooperative sensing [3,19], spectrum sharing [20], channel sensing [21,22] and dynamic channel access [23,24,25]. For example, the authors in [3,19] applied RL to enhance cooperative gain and mitigate cooperative overhead in cognitive radio. The approach eliminates correlated and unreliable cooperative neighboring SUs from cooperation and determines optimal set of cooperative SUs that minimize spectrum sensing delays and control channel traffic. In a bid to improve channel sensing performance, the authors in [26] used channel achievable and channel availability as the basis for determining optimal channel sensing order by applying a low complexity RL algorithm. The approach in [2] achieved energy efficient spectrum sensing by exploration of sensing assignment and exploitation of high throughput frequency bands. Even though the existing RL approaches in the literature made valuable contributions to CR-WSN, none of them considered RL for network clustering

In this paper, we propose an Energy Efficient Spectrum Aware clustering algorithm based on Reinforcement Learning (EESA-RLC) to enhance spectrum hole detection and minimize network energy consumption in CR-WSNs. Reinforcement learning is a machine learning technique that allows an agent to interact with its operating environment and learn an optimal policy that maximizes cumulative rewards [27]. The operating environment can be formulated as a Markov Decision Process (MDP) comprising actions, state of the system, transition rewards, transition probability, performance metric and policy [28]. The agent, which in this case is the SU, detects vacant licensed channels through channel sensing, imposes pairwise constraints to select a clusterhead among the neighboring clusterheads, cooperates with other member nodes in the cluster to determine channel availability, and then chooses an optimal policy that enhances spectrum hole detection and minimizes network energy consumption. The agent employs a Temporal Differences (TD) learning technique [29] to learn from the neighboring clusterheads, evaluates its local decision accuracy, distance to the clusterheads which translates into energy consumption and selects an optimal action policy that optimizes its performance in determining an optimal clusterhead. Although RL is a well-known machine learning technique and has been extensively applied to many fields such as cooperative sensing [3,30], channel sensing sequence [26,22], energy efficient communication [2,31], its application in clustering algorithm is still at the infancy stage. Therefore, our contributions in this paper can be summarized as follows:

- We propose a novel energy-efficient clustering algorithm that is aware of the dynamic radio environment and allows member nodes to learn an optimal policy for choosing optimal clusters based on local decision accuracy and energy consumption for cooperative sensing and data communication.
- The proposed algorithm implements pairwise constraints in spectrum-aware clustering such that only SUs with at least one common vacant channel with a clusterhead and within the clusterhead’s one hop radio range can form a cluster.
- We model network energy consumption, cooperative channel sensing, inter-cluster and intra-cluster data communication energy consumptions and determine an optimal number of network clusters that minimizes network energy consumption.
- We show the performance improvements of the proposed clustering algorithm over Groupwise constraint-based algorithms [17,18] in terms of energy efficiency, channel sensing performance and computational complexity, which make it more attractive for resource constrained devices such as CR-WSNs. In addition, the algorithm eliminates network instability due to re-clustering when the SUs detect PUs’ arrival.

## 2. System Model

**Figure 1:**
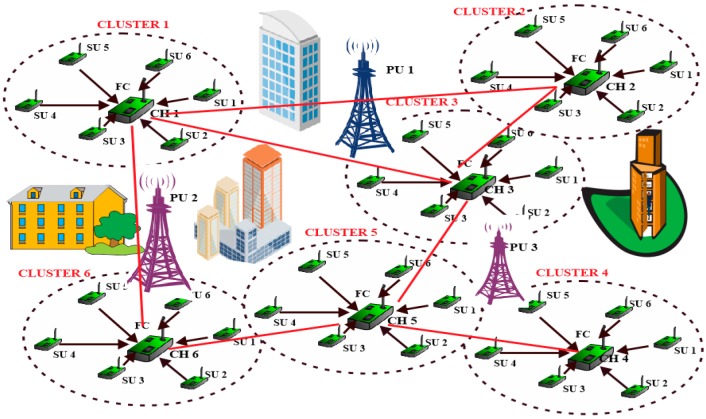
Clustered cooperative channel sensing.

This section describes the system model and parameters adopted for this research. The network is assumed to be static, consisting of N non-mobile homogenous fully functional cognitive radio sensor nodes capable of performing complex tasks. The number of SUs in the network exert a significant impact on energy consumption and on sensing performance. For instance, in a fixed size cluster, the cooperative probability of detection increases along with the increase in the number of cooperative SUs. The network area is partitioned into q clusters, each cluster can be seen as a small cell network comprising a clusterhead and a few member nodes, as shown in Figure 1. Partitioning the network area into clusters has a significant effect on the network energy consumption. If the number of clusters in the network is too small, spectrum sensing, reporting and data communication consume a large amount of energy owing to large number of member nodes per cluster. On the other hand, if the number of clusters is too many, the number of member nodes would be too small and hence the energy consumption per cluster would be low but a large amount of energy would be consumed for inter-cluster communication. Therefore, the optimal number of clusters is extremely important. 

The nodes are uniformly distributed in a two-dimensional square area ${\text{N}}_{\text{A}}$ of L × L square meters and each node is battery powered. This means sensor nodes’ energy cannot be recharged, therefore, nodes’ energy consumption need to be minimized to extend the lifetime of the network. Each node can operate either as a clusterhead or member node. The member nodes MNs sense a set of licensed channels to detect vacant channels, report local sensing decisions to clusterheads for cooperative decision-making and also sense the environment to detect events. The clusterheads perform additional tasks which include decision fusion on the sensing results, controlling access to free channels for data communication and coordinating channel sensing. These additional tasks drain more energy from the battery of the clusterhead, therefore the role of clusterhead will be reassigned to other member nodes within a cluster when the energy depletes below a threshold. It is further assumed that all member nodes lie within the radio range of their respective clusterheads $\left(d<R_{max}\right)$ and communicate directly with the clusterheads in a single-hop manner. This means that member nodes require only low transmissions power and at least one common vacant channel to communicate their data to clusterheads since the distance between them is short.

The operation of the SUs are divided into time slots τ of durations, $\tau^{cs}$ is the time allocated for sensing channels, $\tau^{rp}$ is the time duration for reporting the results, and $\tau^{dt}$ is duration over which the SU can access the free channel for data transmission. There are $n_{z}$ heterogeneous licensed channels in which each channel may exhibit different bandwidth and channel conditions. Larger bandwidth requires longer sensing time which translates into higher energy consumption and poor channel conditions resulting in inaccurate local decisions. An energy detection technique is employed to detect the presence of PUs on the licensed channels, since the PUs’ signals are assumed to be unknown, coupled with the unique resource constraint features of the CR-WSN which requires less complex spectrum sensing techniques.

## 3. The Proposed Energy Efficient Spectrum Aware Reinforcement Learning Based Clustering (EESA-RLC) Algorithm

This section presents modelling and algorithms for our energy-efficient reinforcement learning-based clustering scheme. The algorithms basically comprise of initialization, set-up and coordination phases.

The initialization phase precedes the reinforcement learning process; it involves election of clusterheads. Each SU senses a predefined set of channels to detect the presence or absence of PUs in the channels, computes its clusterhead probability $p_{n}^{ch}$ based on the number of vacant channels detected $\varphi_{n}$ and residual energy ${\text{E}}_{n}^{\text{Re}}$, as well as the required percentage of clusterheads ψ (e.g., 5%) for the network. The clusterhead probability is the probability of a sensor node to become a clusterhead. The main goal is to ensure that all SUs in the network are covered by a set of clusterheads at the initial stage, so that member nodes can directly communicate with clusterheads within their radio range via a single-hop while the clusterheads communicate with the BS through a single-hop or multi-hop fashion. The clusterhead probability $P_{ch}$ for secondary user $SU_{n}$ can be expressed as:

(1) $$p_{n}^{ch}=\frac{{\text{E}}_{n}^{\text{Re}}\;\varphi_{n}\;\psi }{{\text{E}}_{n}^{\text{mx}}\;n_{z}}$$

where ${\text{E}}_{n}^{\text{mx}}$ denotes the reference maximum energy of the SU when fully charged. This clusterhead probability is similar to the HEED protocol described in [32]. 

The clustering initialization process begins at time $\tau_{ini}$. Each SU determines its clusterhead probability and compares it with a given threshold ψ<1. If its clusterhead probability is greater or equal to the given threshold $p_{n}^{ch}\geq \psi$, then the SU emerges as a tentative clusterhead and then broadcasts an advertisement packet $A_{pkt}$ comprising its ID and clusterhead probability. This means that SUs with the highest probability are more likely to emerge as the tentative clusterheads. SUs with clusterhead probability less than the threshold $\left(p_{n}^{ch}<\psi \right)$ hearing the clusterheads announcement withdraw from competing and wait for the final clusterheads announcement by the BS. These SUs are more likely to remain as member nodes while the other in the set perform $N_{ir}=1/\xi$ maximum number of iterations and compete for the role of clusterhead after expiration of announcement waiting period $\sigma_{s}$. Where ξ denotes percentage of the threshold which can be set to a value less than one ξ<1.

In each iteration $i_{tr}$, $i_{tr}\leq N_{ir}$, each of the SUs increases its clusterhead probability $P_{ch}$ by ξ (e.g., ξ = 10% of ψ and compares the updated clusterhead probability with the given threshold. If the clusterhead probability is greater than or equal to the threshold, then it terminates the iteration and broadcasts an advertisement packet. Otherwise, it proceeds to the next iteration. The set of SUs with least number of iterations would emerge as the tentative clusterheads, then after expiration of announcement waiting period $\sigma_{s}$ the other sets that follow them would be elected. This process continues until all nodes are covered by the clusterheads. This means that after the emergence of the first set of clusterheads, each subsequent emergence of sets of clusterheads would be delayed by some duration depending on the number of iterations. The BS selects an optimal number of clusterheads among the tentative clusterheads and broadcasts the list.

The set-up phase mainly deals with cluster formation, based on the advertisement packet $A_{pkt}$ received from multiple neighboring clusterheads $CH_{j}|j=1,\;2,\;3,\ldots ,n_{hg}$ which also denotes clusters $cl_{j}|j=1,\;2,\;3,\ldots ,n_{hg}$, hence clusterhead and cluster will be used interchangeably in this paper. The SU learns the energy consumed and local decision accuracy for each of the clusters by executing model-free reinforcement learning and then selects an optimal cluster $cl_{j}^{*}$ that minimizes energy consumption and enhances spectrum holes detection. During the learning process, the SU senses set of licensed channels $S_{ch}^{t}=\left\{Ch_{z}|z=1,\;2,\;3,\ldots ,n_{z}\right\}$ at every episode t, sends its local decision $LD_{i}^{t}=\;\left\{D_{z}|z=1,\;2,\;3,\ldots ,n_{z}\right\}$ to the clusterhead $CH_{j}$ for the final cooperative decision $CD_{j}^{t}=\;\left\{D_{z}|z=1,\;2,\;3,\ldots ,n_{z}\right\}$ and then compares its local decision $LD_{i}^{t}$ with the cooperative decision $CD_{j}^{t}$ to determine the local decision accuracy $LDA_{j}^{t}$ in respect to cluster $cl_{j}$. It also determines the energy consumption for communicating data $E_{dcost,j}^{t}$ and cooperative sensing $E_{pcost,j}^{t}$. In addition, during the process, favourable clusters which offer minimum energy consumption and better spectrum hole detection would be selected, while excluding the less favorable clusters.

The maintenance phase involves coordination of cluster members. In this phase, the clusterheads specify the set of channels $S_{ch}$ to be sensed based on their availabilities and control access to the free licensed channels for data communication. Upon energy depletion of any clusterhead, the clusterhead initiates re-clustering process and a new clusterhead would emerge among the member nodes.

The main objective of the algorithm is to achieve an optimal policy for selecting optimal cluster or clusterhead that satisfies the pairwise constraint conditions, minimizes cooperative channel sensing energy consumption and data communication energy consumption while enhancing spectrum hole detection. 

Let $EH=\left\{CH_{j}|j=1,\;2,\;3,\ldots ,hn\right\}$ denote a set of clusterheads and $H_{n}=\left\{CH_{j}|j=1,\;2,\;3,\ldots ,n_{hg}\right\}$ denote a set of neighbouring clusterheads such that $H_{n}\subset EH$. And let $CL_{n}=\left\{cl_{j}|j=1,\;2,\;3,\ldots ,n_{hg}\right\}$ and $E_{TC}^{set}\left(B_{dt},d\right)=\left\{E_{TC,j}\text{|}j=1,\;2,\;3,\ldots ,n_{hg}\right\}$ denote the corresponding set of neighbouring clusters and set of energy consumed for transmitting $B_{dt}$-bits data packet to the respective clusterheads at distance d and for cooperative sensing of set of channels $S_{ch}=\left\{Ch_{z}|z=1,\;2,\;3,\ldots ,n_{z}\right\}$. Each cluster $cl_{j}$ consists of a clusterhead $CH_{j}$ and member nodes $MN_{i}\text{|}i=1,\;2,\;3,\ldots ,m_{n}\text{]}$ such that $cl_{j}=\left\{CH_{j},MN_{i}|i=1,\;2,\;3,\ldots ,m_{n}\right\}$. If $M_{V,i}=\left\{Ch_{z}|z=1,\;2,\;3,\ldots ,n_{mv}\right\}$ and $H_{V,j}=\left\{Ch_{z}|z=1,\;2,\;3,\ldots ,n_{hv}\right\}$ denote sets of vacant channels detected by the member node $MN_{i}$ and the selected clusterhead $CH_{j}$, respectively, then the problem of finding optimal clusterhead $CH_{j}^{*}$ can be formulated as a Markov Decision Process while the energy minimization problem can be formulated as pairwise constraint Sum of Square Error (SSE) minimization problem subject to one hop transmission constraint. This is given as:

(2a) $$\arg min\overset{q}{\underset{j=1}{\sum }}\overset{m_{n}}{\underset{i=1}{\sum }}d^{\beta }\left(i,j\right)$$

Subject to:

(2b) $$\begin{array}{c} \text{C1:}M_{V,i}\cap H_{V,j}\neq \emptyset \\ \text{C2:}\begin{array}{c} \max d\left(i,j\right)<R_{max}\\ MN_{i},CH_{j}\epsilon cl_{j} \end{array} \end{array}$$

To achieve this, Q-learning would be adopted due to its model-free capability and the pairwise constraint would be applied on the member nodes and the clusterheads during the clustering.

### 3.1. Pairwise Constraint Clustering

**Figure 2:**
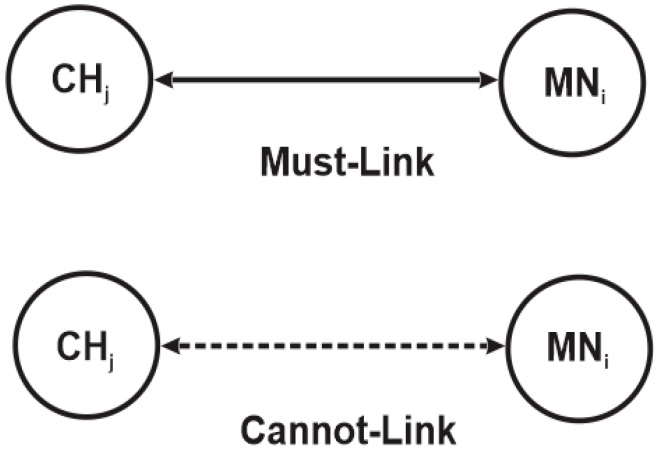
Illustration of must-link and cannot-link constraints.

The concept of pairwise constraints has been widely implemented in many clustering algorithms such as k-means [33] and complete link [34] clustering to impose must-link and cannot-link constraints on pairs of nodes during the clustering as illustrated in Figure 2. The must-link constraint forces pair nodes $n_{a}$ and $n_{b}$ to be placed in the same cluster, while the cannot-link constraint disallows pair nodes $n_{a}$ and $n_{b}$ to be placed in the same cluster [17]. This significantly influences the outcome of the clustering, since pair nodes with common links usually belong to the same cluster, while those without common links belong to different clusters. 

**Figure 3:**
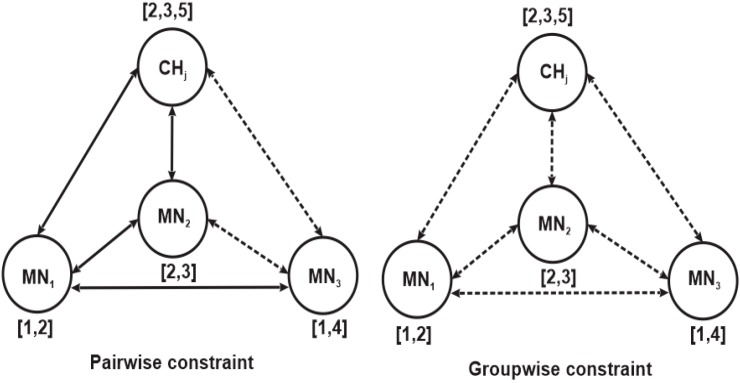
Illusration of pairwise and groupwise constraints.

Therefore, the pairwise constraint concept can also be implemented in a spectrum-aware clustering algorithm, which can be explained by comparing it with the groupwise constraint method employed in [17,18] for spectrum-aware clustering. Unlike conventional WSN clustering schemes, spectrum-aware clustering schemes require each node to sense the spectrum band and detect spectrum holes that can be used for data communication. In addition, each clusterhead must have at least one common available channel with its member nodes since each member node transmits its data directly to the clusterhead without intermediary nodes. To highlight the difference between pairwise and groupwise constraints, we consider a clusterhead $CH_{j}$ and three member nodes $MN_{1}$, $MN_{2}$, $MN_{3}$ that operate on a set of licensed channels $S_{ch}=\left\{Ch_{z}|z=1,\;2,\;3,\;\ldots ,\;n_{z}\right\}$ as shown in Figure 3. The numbers beside them represent the vacant licensed channels detected by the respective member node; a dotted line between them indicates a cannot-link constraint, while a solid line indicates a must link constraint as illustrated in Figure 2. The figure shows that member node $MN_{1}$ shares channel $Ch_{2}$ with $MN_{2}$ and $CH_{j}$, member node $MN_{2}$ shares channel $Ch_{2}$ and channel $Ch_{3}$ with $CH_{j}$ in addition to member node $MN_{1}$, while member node $MN_{3}$ shares only channel $Ch_{1}$ with $MN_{1}$. Based on this scenario, the pairwise constraint imposes a must-link constraint on member nodes $MN_{1}$ and $MN_{2}$ to form a cluster with clusterhead $CH_{j}$ because they all share a vacant channel ($Ch_{2}$). It also imposes a cannot-link constraint on $CH_{j}$ and $MN_{3}$ so that the member nodes cannot form a cluster since member node $MN_{3}$ has no common vacant channel with $CH_{j}$ even though it shares a vacant channel with member node $MN_{1}$. On the other hand, the groupwise constraint imposes cannot-links on all the four nodes, including $CH_{j}$, disallowing them from forming a cluster, because the nodes share no common vacant channel. While a pairwise constraint requires at least one common vacant channel for pair nodes of clusterhead and member node to form a cluster, a groupwise constraint requires at least one common vacant channel for all nodes in a group to a form cluster.

### 3.2. Cooperative Channels Sensing

Spectrum sensing is a key function of cognitive radio for determining licensed channel occupancy; this is done by detecting the existence of PUs in the channels. Energy detection technique has been commonly employed to detect the existence of PUs’ signals in the spectrum bands by measuring energy of the received signal waveform over a specified observation time. The received signal is first filtered by a Band Pass Filter (BPF) to limit the noise bandwidth. The filtered output signal of bandwidth $B_{w}$ is converted to discrete samples $N_{o}$ by an Analogue-to-Digital Converter (ADC) and then passed through an integrator for an observation interval τ. The final average energy of the observed samples $N_{o}$ from the output of the integrator $Y=\overset{N_{o}}{\underset{\tau =1}{\sum }}{\left|y\left(\tau \right)\right|}^{2}$ is compared with a threshold λ to determine the existence or otherwise of a PU signal [4]. If Y<λ, then a PU’s signal is absent and the channel is considered available, otherwise, a PU’s signal is considered to be present and the channel is being occupied. Thus, the received signal at the SU Y can be expressed as [4]:

(3) $$Y=\;\{\begin{array}{c} x\left(\tau \right),\;H_{o}\\ z\left(\tau \right)+x\left(\tau \right),\;H_{1} \end{array}$$

where x(τ) denotes zero-mean Additive White Gaussian Noise (AWGN), z(τ) denotes the received signal waveform. $H_{o}$ denotes the null hypothesis which indicates the absence of a PU’s signal, while $H_{1}$ denotes a hypothesis which indicates the presence of a PU’s signal. Thus, the test statistics Y from the output of the integrator follow a chi-square distribution and can be approximated to a Gaussian distribution using central limit theorem, which when the number of samples is large given as [29]:

(4) $$Y\approx \{\begin{array}{c} N\left(N_{o}\sigma_{n}^{2},\;2n\sigma_{n}^{4}\right),\;H_{0}\;\\ N\left(N_{o}\left(\sigma_{n}^{2}+\sigma_{z}^{2}\right),\;2N_{o}{\left(\sigma_{n}^{2}+\sigma_{z}^{2}\right)}^{2}\right),\;H_{1} \end{array}$$

where $\sigma_{n}^{2}$ denotes received the noise signal’s variance and $\sigma_{z}^{2}$ denotes the received signal’s variance z(τ). Optimal PU detection can be achieved through a Maximum *A Posteriori* (MAP) scheme which models the PU behaviors into On and Off states using a two-state Markov chain that has been widely adopted [35,36,37]. Therefore, the probability of detection $P_{d}$ which suggests the presence of a PU in the considered channel can be expressed as:

(5) $$\begin{array}{l} P_{d}(\lambda )=P_{r}\left[Y>\lambda \backslash H_{1}\right]P_{on}\\ =\mathbb{Q}\left(\frac{\lambda -2B_{w}T\left(\sigma_{n}^{2}+\sigma_{z}^{2}\right)}{\sqrt{4B_{w}T{\left(\sigma_{n}^{2}+\sigma_{z}^{2}\right)}^{2}}}\right)\;.\;P_{on} \end{array}$$

Similarly, the probability of false alarm $P_{f}$ which falsely indicates the presence of a PU’s signal in the considered channel can be expressed as:

(6) $$\begin{array}{l} P_{f}\left(\lambda \right)=P_{r}\left[Y>\lambda \backslash H_{0}\right]P_{off}\\ =\mathbb{Q}\left(\frac{\lambda -2B_{w}T\sigma_{n}^{2}}{\sqrt{4B_{w}T\sigma_{n}^{4}}}\right).\;P_{off} \end{array}$$

where ℚ(.) is the generalized Marcum Q-function, $P_{on}$ and $P_{off}$ are probabilities that the channel is in busy or idle states respectively.

Cooperative spectrum sensing enhances the PU detection through exploitation of SUs’ observed signals spatial diversity. Each of the $MN_{i}$ senses set of channels $S_{ch}=\left\{Ch_{z}|z=1,\;2,\;3,\ldots ,n_{z}\right\}$, makes a local decision $LD_{i\;}=\;\left\{D_{z}|z=1,\;2,\;3,\ldots ,n_{z}\right\}$ on the existence of PUs in the channels or otherwise and then reports its sensing result to the cluster head $CH_{j}$ for decision fusion and final cooperative decision $CD_{\text{j}}\;=\;\left\{D_{z}|z=1,\;2,\;3,\ldots ,n_{z}\right\}$. Local decision $D_{z}=0$ indicates the presence of a PU’s signal in the observed channel $Ch_{z}$, while $D_{z}=1$ denotes the absence of a PU’s signal in the considered channel $Ch_{z}$. It is assumed that the channel between $MN_{i}$ and $CH_{j}$ is a perfect channel since the distance between them is short. The clusterhead $CH_{j}$ employs “M-out-of-N majority” decision counting rule fusion to determine the existence of PU in the channels $S_{ch}=\left\{Ch_{z}|z=1,\;2,\;3,\ldots ,n_{z}\right\}$ and then broadcasts the outcome. The final cooperative decisions $CD_{j}$ based on this rule indicates the presence of a PU’s signal in the channel when ℓ out of m sensing results indicate the presence of a PU’s signal in the channel [38]. This implies that when the number of cooperative member nodes $cm_{n}$ that report presence of PU’s signal in the considered channel is greater than or equal to half of the total number of cooperative member node ($cm_{n}\geq m_{n}/2)$, then the final cooperative decision indicates the presence of a PU in the observed channels [39]. Otherwise it indicates the absence of a PU and hence the availability of the observed channels. Thus, the cooperative probability of detection $Q_{d,j}$ is given as [30]:

(7) $$Q_{d,j}=\overset{m}{\underset{\;\ell =n}{\sum }}\left(\begin{array}{c} m\\ \ell \end{array}\right){\text{P}}_{d}^{\ell }{\left(1-P_{\text{d}}\right)}^{m-\ell }\;$$

### 3.3. Cognitive Radio Wireless Sensor Network Energy Consumption Model

Network energy consumption for CR-WSNs mainly comprises of energy consumptions for vacant channels detection, event sensing, data processing, and communication. The energy consumption for detecting vacant channels is the energy consumed for cooperative channels sensing. The event sensing energy consumption is attributed to energy consumed for the sensing event while the data processing energy is attributed to energy consumed for data logging. The energy consumption for data transmission is attributed to energy consumed for intra-cluster and inter-cluster data communications. 

Several attempts have been made to model wireless sensor network energy consumptions. The most widely adopted models [40,41,42] have combined the impact of the external radio environment and the sensor node’s communication hardware together. According to these models, energy consumption for transmitting a unit bit of data depends largely on the distance between the transmitting and the receiving nodes. However, the outcome of experimental measurements conducted in [43] is inconsistent with the widely adopted simplified models. In addition, it is revealed that separating energy consumption of each hardware component from the external radio environment may lead to a more realistic energy consumption model [44]. The authors in [45] proposed a comprehensive node power consumption model which considered other energy consumption sources that were ignored in the previous models such as sensor sensing and sensor logging. However, these approaches are specifically developed for conventional sensor nodes without due consideration of the cognitive radio aspect. Therefore, accurate estimation of CR-WSN life expectancy requires a realistic network energy consumption model that incorporates channel sensing energy consumption as well. 

**Figure 4:**
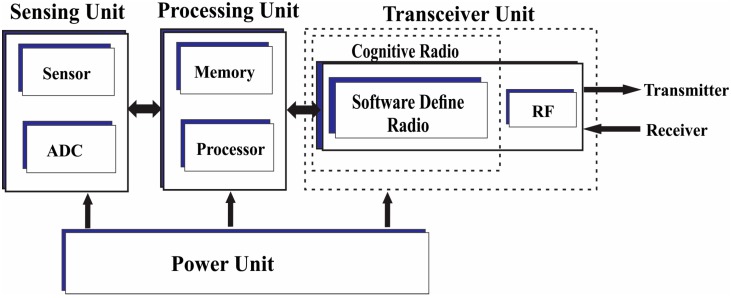
Block diagram of a cognitive radio sensor node.

The main components of a cognitive radio sensor node are event sensing, data processing, communication and cognitive radio units as shown in Figure 4. The event sensing unit monitors the environment and generates signal traffic whenever an event is occurred. The processing unit processes the data while the communication unit transmits the data to desire sink over a free licensed channel and also receives data. The cognitive radio module detects set of unused licensed channels and then accesses the most suitable channel to communicate the data.

#### 3.3.1. Event Sensing Unit Energy Consumption

The event sensing unit interacts with the physical environment to detect an event and then convert the physical signals to digital signals. Signal sampling, physical signal conversion to electrical signals and analogue signal to digital signal conversion are the main sources of energy consumption in the event sensing unit [45]. Let $T_{ss}$ denotes the event sensing duration, $P_{ss}$ denotes power required for the event sensing activity which includes event detection and signal conversion and $B_{ss}$ denotes the bits packet. The energy dissipation for event sensing activity for $B_{ss}\;$bits packet is given as:

(8) $$E_{ss}\left(B_{ss}\right)=P_{ss}\;T_{ss}\;B_{ss}\;$$

#### 3.3.2. Processing Unit Energy Consumption

The processing unit executes functions such as sensor data logging, data aggregation and processing. Energy consumption for sensor data logging is due to amount of energy consumed for reading and writing a packet of data into the memory. Let $P_{rd}$ denote the power consumption for reading a packet from the memory, $P_{wr}$ denotes power consumption for writing a packet into the memory then, the energy consumption for data logging can be expressed as [45]:

(9) $$E_{log}\left(B_{lg}\right)=B_{lg}\left(P_{rd}+\;P_{wr}\right)$$

Energy consumption for data aggregation and processing are mainly derived from microcontroller energy losses which occur as a result of switching and leakage currents. The total energy consumption for processing or aggregating $B_{ap}$ bits of data packet can be expressed as [46]:

(10) $$E_{ap}\left(B_{ap},N_{cy}\right)=B_{ap}N_{cy}C_{av}V_{s}^{2}+B_{ap}V_{s}\left(I_{o}e^{\frac{V_{s}}{n_{p}V_{t}}}\right)\left(\frac{N_{cy}}{f}\right)$$

where $N_{cy}$ denotes the number of clock cycles per task, $I_{o}$ denotes the leakage current, $C_{av}$ denotes average number of capacitance switches per cycle, f denotes the frequency of the sensor, $n_{p}$ denotes a constant parameter defined by the processor, $V_{s}$ and $V_{t}$ denote the source and terminal voltage, respectively [45].

#### 3.3.3. Cognitive Radio Unit Energy Consumption

The CR unit senses the licensed channels and detects vacant channels that can be used for data communications. Energy consumption for vacant channels detection comprises energy consumption for sensing sets of channels and reporting local decisions as well as receiving final cooperative decisions [47]. Energy consumption for sensing sets of channels ${\text{E}}_{\text{cs}}$ comprises of energy consumed for listening over the channels and receiving $N_{o}$ observation samples, as well as energy required to process the signal samples (modulation, signal shaping *etc.*) and make local decisions. If $P_{ed}$ denotes the energy detector’s circuit power consumption and $E_{sp}$ denotes the energy consumption for processing the received $N_{o}$ signal samples, then energy dissipation for sensing $n_{z}$ sets of channels can be expressed as:

(11) $${\text{E}}_{\text{cs}}\left(n_{z},T_{cs}\right)=\sum_{m=1}^{\;n_{z}}\left(T_{cs}\;P_{ed}+{\text{E}}_{sp}\right)$$

This suggests that the energy consumption for channel sensing is a function of channel sensing duration $T_{cs}$ and it increases along with an increase in the number of channels $n_{z}$. Minimum energy consumption can be achieved with minimum channel sensing time but accurate results may not be obtained. The Nyquist sampling theorem suggests that the sample frequency $f_{s}\;$of the received signal samples ($N_{o}=2T_{cs}B_{w})$ must be at least twice the bandwidth ($f_{s}\geq 2B_{w}$) [29]. Let $P_{dt}$ denote the target probability of detection, $P_{ft}$ denotes target probability of false alarms, and $\delta_{s}$ denotes the average of the PU’s SNR received on the channel. The channel sensing time $T_{cs}$ can be expressed as [37]:

(12) $$T_{cs}=\frac{1}{{\delta_{s}}^{2}f_{s}}{\left(Q^{-1}\left(P_{ft}\right)-Q^{-1}\left(P_{dt}\right)\sqrt{2\delta_{s}+1}\right)}^{2}\;$$

In cooperative sensing, each member node $MN_{i}$ senses sets of channels to detect vacant channels, makes local decisions on the existence of PU and then reports its result to the FC which is the clusterhead $CH_{j}$ for the final cooperative decision.

Let $d_{i,j}$ denote the Euclidian distance between $MN_{i}\;$and $CH_{j}$, $E_{ec}$ denotes the energy consumption for running the radio electronics of $MN_{i}$ and $E_{am}$ denotes the energy consumption for amplifying the signal to be transmitted to $CH_{j}\;$so as to maintain an acceptable SNR level. Then the energy cost $E_{rp}$ for reporting $B_{ld}$-bits packet of local decisions $\text{to the }CH_{j}$ can be expressed as:

(13) $${\text{E}}_{rp}\left(B_{ld},d_{i,j}\right)=\;B_{ld}\left(E_{ec}+E_{am}\;d_{i,j}\right)\;$$

The energy cost for receiving the $B_{ld}$ bits packet of final cooperative decision broadcast by the $CH_{j}$ after performing a decision is mainly determined by the number of bits in the packet and energy consumed for running the radio electronics circuitry. Therefore, the energy consumption for receiving $B_{ld}$-bits of broadcasted packet can be expressed as:

(14) $${\text{E}}_{rx}\left(B_{ld}\right)=\;B_{ld}E_{ec}$$

Therefore, the energy consumed by member node $MN_{i}$ is the energy consumption for cooperative channel sensing which comprises the energy consumption for sensing the set of channels, energy consumption for reporting local decisions and energy consumption for receiving the final cooperative decision which is given as:

(15) $$E_{MN}^{cs}=\underset{\begin{array}{c} Channels\;\\ Sensing \end{array}}{\underset{︸}{{\text{E}}_{\text{cs}}\left(n_{z},T_{cs}\right)}}+\underset{\begin{array}{c} Decision\;\\ Reporting \end{array}}{\underset{︸}{{\text{E}}_{rp}\left(B_{ld},d_{i,j}\right)}}+\underset{\begin{array}{c} \;Coop.\;Decision\\ Receiving \end{array}}{\underset{︸}{{\text{E}}_{rx}\left(B_{cd}\right)}}$$

Each $CH_{j}$ performs data fusion upon receiving MNs’ local decisions and then broadcasts the final cooperative decision. Energy consumption ${\text{E}}_{rx}$ for receiving $B_{ld}$-bits of each local decision is given as:

(16) $${\text{E}}_{rx}\left(B_{ld}\right)=\;B_{ld}E_{ec}\;$$

Let $R_{max}$ denote the maximum radio range of clusterhead $CH_{j}$, then the consumed energy ${\text{E}}_{bd}$ for broadcasting the final cooperative decision can be expressed as:

(17) $${\text{E}}_{bd}\left(B_{ld},d_{j,R_{max}}\right)=\;B_{ld}\left(E_{ec}+E_{am}\;d_{j,R_{max}}\right)$$

Let ${\text{E}}_{dp}$ denote energy consumption for processing a $B_{ld}$ bits packet received from each member node $MN_{i}$ for decision fusion. Energy cost for clusterhead $CH_{j}$ cooperative channel sensing is the energy consumed for sensing a set of channels, energy consumed for receiving $m_{n}$ member nodes’ local decisions, energy consumption for processing the decisions and energy consumption for broadcasting the final cooperative decisions which is given as:

(18) $$E_{CH}^{cs}=\underset{\begin{array}{c} Channels\;\\ Sensing \end{array}}{\underset{︸}{{\text{E}}_{\text{cs}}\left(n_{z},T_{cs}\right)}}+\underset{\begin{array}{c} Decision\\ Receiving\; \end{array}}{\underset{︸}{\overset{m_{n}}{\underset{e=1}{\sum }}{\text{E}}_{rx}\left(B_{ld}\right)}}+\underset{\begin{array}{c} \;Decision\\ Processing \end{array}}{\underset{︸}{\overset{m_{n}+1}{\underset{e=1}{\sum }}{\text{E}}_{dp}\left(B_{ld}\right)}}+\underset{\begin{array}{c} Coop.Decision\\ Broadcasting \end{array}}{\underset{︸}{{\text{E}}_{bd}\left(B_{ld},d_{j,R_{max}}\right)}}$$

Therefore, total energy cost for cooperative channel sensing $E_{total}^{cs}$ can be expressed as:

(19) $$\begin{array}{c} E_{total}^{cs}=\overset{k}{\underset{j=1}{\sum }}\left(\overset{m_{n}}{\underset{e=1}{\sum }}E_{MN}^{cs}+E_{CH}^{cs}\right)\\ =\overset{k}{\underset{j=1}{\sum }}\left(\overset{m_{n}}{\underset{e=1}{\sum }}\left(E_{rp}\left(B_{ld},d_{i,j}\right)+2E_{rx}\left(B_{cd}\right)\right)\;+\overset{m_{n}+1}{\underset{e=1}{\sum }}\left(E_{cs}\left(n_{z},T_{cs}\right)+E_{dp}\left(B_{dp}\right)\right)+E_{bd}\left(B_{cd},d_{j,R_{max}}\right)\right) \end{array}$$

#### 3.3.4. Transceiver Unit Energy Consumption

The transceiver unit enables communication between the member nodes MNs and the clusterheads CHs as well as between the CHs and the BS. Each $MN_{i}$ transmits its reading data to any of the selected clusterheads $CH_{j}$ through the available licensed channels. Since all MNs are within the radio range of their neighboring clusterheads, adjacent MNs can send their data to the $CH_{j}$ without intermediary nodes. Energy consumption ${\text{E}}_{tM}$ for transmitting $B_{dt}$-bits packet to $CH_{j}$ over a distance $d_{i,j}$ can be expressed as:

(20) $${\text{E}}_{tM}\left(B_{dt},d_{i,j}\right)=\;B_{dt}\left(E_{ec}+E_{am}\;d_{i,j}\right)\;$$

The $CH_{j}$ aggregates the data received from the MNs and then forwards the aggregated data either through some intermediate neighbouring clusterhead $CH_{g}$ or directly to the BS. Energy consumption ${\text{E}}_{rM}$ for receiving $B_{dt}$-bits packet from member node $MN_{i}$ can be expressed as:

(21) $${\text{E}}_{rM}\left(B_{dt}\right)=\;B_{dt}E_{ec}\;$$

Let $CH_{g}$ denote the immediate neighbouring clusterhead through which the aggregated data packets $B_{pk}$ will be routed to the BS, $E_{mp}$ denotes the energy consumption for amplifying the signal and $d_{j,g}$ denotes the distance between clusterhead $CH_{j}\;$and the immediate neighbouring clusterhead $CH_{g}$ or the BS. The energy consumption ${\text{E}}_{tH}$ for transmitting the aggregated data packets $B_{pk}$ to the immediate neighboring clusterhead $CH_{g}$ or the BS over a distance $d_{j,g}$ can be expressed as:

(22) $${\text{E}}_{tH}\left(B_{pk},d_{j,g}\right)=\;B_{pk}\left(E_{ec}+E_{mp}\;d_{j,g}\right)$$

Energy consumption for receiving the aggregated data packets $B_{pk}$ from neighbouring clusterhead $CH_{g}$ for onward transmission is given as:

(23) $${\text{E}}_{rH}\left(B_{pk}\right)=\;B_{pk}E_{ec}\;$$

The source of power consumption in the RF-front end is mainly dominated by power amplifier which boosts the transmission power to a certain level depending on the type of the amplifier and the application. The power amplifier’s power consumption $P_{am}$ which is a function of transmission distance d largely depends on many factors which include operating frequency, DC supply voltage, output power, hardware technology and load characteristic. The total power consumption for running the power amplifier is equal to the DC input power $P_{dc}$($P_{am}$ = $P_{dc}$) [44]. The ability of the power amplifier to convert the DC input power $P_{dc}$ into RF signal power $P_{tx}\;$is referred to as drain efficiency. This efficiency can be expressed as the ratio of signal power to DC power given as:

(24) $$\eta =\frac{P_{tx}}{P_{dc}}\;$$

Communication over the wireless medium is susceptible to propagation impairments such as multi-path, fading and attenuation. If $P_{tx}$ denotes the RF signal power from the transmitter’s power amplifier delivered to the receiver node’s antenna, and G is a parameter that defines the characteristic of the transmitting and receiving antenna, then the RF signal power $P_{rx}$ received at the receiving sensor node can be expressed as:

(25) $$P_{rx}=\frac{P_{tx}}{\left(G\;d^{\beta }\right)}$$

Therefore, from Equations (24) and (25), the minimum power consumption of RF power amplifier to amplify transmission signals is given by $P_{am}\;=\mu \;/\eta$, where $\mu =P_{rx}\;G\;$is a constant given by the received RF signal power ${\text{P}}_{\text{rx}}$ and the antenna characteristic G [44]. The parameter value μ is a function of radio environment and can be set to a single-hop maximum transmission power value *i.e.*, 6.3 mW instead of absolute value as in [44]. The energy consumption for data transmission comprises of energy cost for intra-cluster and inter-cluster data communication. 

In intra-cluster data communication, distance between the MNs and their prospective CHs is presumably short and therefore, the channel between them follows the Friis free space path loss model with signal power attenuation of β=2 power loss [30]. Thus, energy consumption for intra-cluster communication comprises of total energy consumption of all MNs for transmitting $B_{dt}$ bits of data packet over a distance $d_{i,j}$ to their respective clusterheads $CH_{j}$ and total energy consumption for receiving the data by the clusterhead $CH_{j}$. This is given as:

(26) $$E_{Intr}\left(i,j\right)=\;\overset{\text{q}}{\underset{\text{j}=1}{\sum }}\overset{{\text{m}}_{n}}{\underset{\text{i}=1}{\sum }}\left(\underset{\begin{array}{c} \;Data\\ Transmitting \end{array}}{\underset{︸}{{\text{E}}_{tM}\left(B_{dt},d_{i,j}\right)}}+\underset{\begin{array}{c} \;Data\\ Receiving \end{array}}{\underset{︸}{{\text{E}}_{rM}\left(B_{dt}\right)}}\right)\;$$

In inter-cluster data communications, the distance between CHs and BS is presumably long and therefore, the channel between them follows the Friis free space path loss model with signal power attenuation of β=4 power loss [30]. Each clusterhead $CH_{j}$ forwards its aggregated data packets $B_{pk}$ to the BS either through intermediate clusterhead $CH_{g}$ $\left(if\;d_{j,g}>R_{max,j}\right)$ or direct to the BS without any intermediate clusterhead $CH_{g}$ $\left(if\;d_{j,g}\leq R_{max,j}\right)$. The total energy consumption for inter-cluster data communications is the energy consumption for aggregating the received data and energy consumption for forwarding the data packet to the BS which is given as:

(27) $$\;E_{int}=\overset{q}{\underset{j=1}{\sum }}\left(\underset{\begin{array}{c} Data\;\\ Aggregation \end{array}}{\underset{︸}{E_{ap}\left(B_{ap},N_{cy}\right)}}+\underset{\begin{array}{c} Data\;\\ Transmitting \end{array}}{\underset{︸}{{\text{E}}_{tH}\left(B_{pk},d_{j,g}\right)}}\right)$$

Thus, the total energy consumption for data communications can be expressed as:

(28) $$\begin{array}{c} E_{total}^{dt}=E_{Intr}\left(i,j\right)+E_{int}\\ =\overset{q}{\underset{\text{j}=1}{\sum }}\left(\overset{{\text{m}}_{n}}{\underset{\text{i}=1}{\sum }}(\underset{\begin{array}{c} Data\\ Transmitting \end{array}}{\underset{︸}{E_{tM}\left(B_{dt},d_{ij}\right)}}+\underset{\begin{array}{c} Data\\ Receiving \end{array}}{\underset{︸}{{\text{E}}_{rM}\left(B_{dt}\right)}})+\underset{\begin{array}{c} Data\;\\ Aggregation \end{array}}{\underset{︸}{E_{ap}\left(B_{ap},N_{cy}\right)}}+\underset{\begin{array}{c} Data\;\\ Transmitting \end{array}}{\underset{︸}{{\text{E}}_{tH}\left(B_{pk},d_{j,g}\right)}}\right) \end{array}$$

If $m_{n,j}$ denotes the number of member nodes in cluster $cl_{j}$ and q is the number of clusters in the network which is also equal to the number of clusterheads in the network, then the number of cognitive radio sensor nodes in the network $N=\sum_{j=1}^{q}\left(m_{n,j}+1\right)$. Therefore, total energy consumed by member node $MN_{i}$ in cluster $cl_{j}$ is given as:

(29) $$\begin{array}{c} E_{MN}\left(i,j\right)=\underset{\begin{array}{c} Events\\ \;Sensing \end{array}}{\underset{︸}{E_{ss}(B_{ss})}}+\underset{\begin{array}{c} Data\\ Logging \end{array}}{\underset{︸}{E_{log}(B_{lg})}}+\underset{\begin{array}{c} Channel\\ sensing \end{array}}{\underset{︸}{{\text{E}}_{cs}(n_{z},T_{cs})}}+\underset{\begin{array}{c} Decision\\ Reporting \end{array}}{\underset{︸}{{\text{E}}_{rp}(B_{ld},d_{i,j})}}+\underset{\begin{array}{c} Coop.Decision\\ Receiving \end{array}}{\underset{︸}{{\text{E}}_{rx}(B_{ld})}}+\underset{\begin{array}{c} Data\\ Transmitting \end{array}}{\underset{︸}{{\text{E}}_{tM}(B_{dt},d_{ij})}} \end{array}=P_{ss}\;x\;T_{ss}\;x\;B_{ss}+B_{lg}\left(P_{rd}+\;P_{wr}\right)+\overset{\;n_{z}}{\underset{m=1}{\sum }}\left(T_{cs}{\text{P}}_{ed}+{\text{E}}_{sp}\right)+B_{ld}\left(2E_{ec}+E_{am}\;d_{i,j}^{\beta }\right)+B_{dt}\left(E_{ec}+E_{am}\;d_{i,j}^{\beta }\right)\;$$

Similarly, total energy consumption of clusterhead $CH_{j}$ in cluster $cl_{j}$ is given as:

(30) $$\begin{array}{c} E_{CH}(j)=\underset{\begin{array}{c} Events\\ Sensing \end{array}}{\underset{︸}{E_{ss}(B_{ss})}}+\underset{\begin{array}{c} Data\\ Logging \end{array}}{\underset{︸}{E_{\log}(B_{\mathrm{lg}})}}+\underset{\begin{array}{c} Channel\\ sensing \end{array}}{\underset{︸}{{\text{E}}_{cs}(n_{z},T_{cs})}}+\sum_{i=1}^{{\text{m}}_{n}}\left(\underset{\begin{array}{c} Decisions\\ \mathrm{Receiving} \end{array}}{\underset{︸}{E_{rx}(B_{ld})}}+\underset{\begin{array}{c} Data\\ \mathrm{Receiving} \end{array}}{\underset{︸}{E_{rM}(B_{dt})}}\right)\\ +\sum_{i=1}^{{\text{m}}_{n}+1}\left(\underset{\begin{array}{c} Decisions\\ \mathrm{Processing} \end{array}}{\underset{︸}{E_{dp}(B_{dp})}}+\underset{\begin{array}{c} Data\\ Aggregation \end{array}}{\underset{︸}{E_{ap}(B_{dt},N_{cy})}}\right)+\underset{\begin{array}{c} Data\\ Transmitting \end{array}}{\underset{︸}{{\text{E}}_{tM}(B_{dt},d_{i,j})}}\\ =P_{ss}\;x\;T_{ss}\;x\;B_{ss}+B_{\mathrm{lg}}(P_{rd}+P_{wr})+\sum_{m=1}^{n_{z}}\left(T_{cs}\;P_{ed}+E_{sp}\right)+\sum_{i=1}^{{\text{m}}_{n}}\left(B_{ld}\;E_{ec}+B_{dt}\;E_{ec}\right)\\ +\sum_{i=1}^{{\text{m}}_{n}+1}\left(E_{dp}(B_{dp}\;)+B_{ap}N_{cy}C_{av}V_{s}^{2}+B_{ap}V_{s}\left(I_{o}e^{\frac{V_{s}}{n_{p}V_{t}}}\right)\left(\frac{N_{cy}}{f}\right)\right)\\ +B_{cd}\left(E_{ec}+E_{am}\;d_{j,R_{\max}}^{\beta }\right)+B_{pk}P_{ec}+B_{pk}\left(E_{ec}+E_{mp}\;d_{j,g}^{\beta }\right) \end{array}$$

Thus, total energy consumption for the entire network is given as:

(31) $$E_{net}=\sum_{j=1}^{q}\left(E_{CH}\left(j\right)+\sum_{i=1}^{m_{n,j}}E_{MN}\left(i,j\right)\right)$$

### 3.4. Optimal Number of Clusters

Network clustering is a promising technique that can be employed to achieve network scalability, reliable and energy efficient communication. A near-centre member node $MN_{i}$ in a cluster consumes less energy for intra-cluster communication than a near-border member node $MN_{g}$ but both consume maximum power for inter-cluster communication when they are selected as clusterheads [17]. This means a shorter average distance between member nodes and clusterhead requires less energy for intra-cluster communication. Therefore, the number of clusters which influences member nodes distribution in each cluster and average intra-cluster distance are key elements to be considered in minimizing network energy consumption. The optimal number of clusters $q^{*}$ need to be carefully determined so that network-wide energy consumption can be minimized. The network is partitioned into q number of clusters with each cluster comprises of one clusterhead $CH_{j}$ and 1−(Nq) number of member nodes mn≈Nq uniformly distributed within the radio range of the clusterhead $CH_{j}$. Let $A_{sp}^{2}$ denotes average spans for x-axis and y-axis of each cluster $cl_{j}$, $A_{c}$ denotes average cluster area and ρ(x,y) denotes nodes distribution density within a cluster. The Euclidian square distance $d_{i,j}^{2}$ between the member node $MN_{i}\;$and the clusterhead $CH_{j}$ can be expressed as:

(32) $$d_{i,j}^{2}=\iint \left(x^{2}+y^{2}\right)\rho \left(x,y\right)dxdy$$

If area $A_{c,i}$ of cluster $cl_{j}$ is assumed to be a two dimensional area, then the average span $A_{sp}^{2}=L^{2}/q$ and the node distribution density $\rho =q/L^{2}$. Thus, Equation (32) can be expressed as:

(33) $$d_{i,j}^{2}=\rho \int_{x=0}^{L/\sqrt{q}}\int_{y=0}^{L/\sqrt{q}}\left(x^{2}+y^{2}\right)dxdy$$

After further simplification, the equation reduces to:

(34) $$d_{i,j}^{2}\;=\frac{2}{3}\frac{L^{2}}{q}\;$$

Therefore, optimal number of cluster $q^{*}$ can be derived analytically from the network wide energy consumption equation which is given as:

(35) $$\begin{array}{c} E_{net}=\sum_{j=1}^{q}\left(E_{CH}(j)+\sum_{i=1}^{m_{n,j}}E_{MN}(i,j)\right)\\ =\sum_{j=1}^{q}\left(E_{ss}+E_{log}+{\text{E}}_{\text{cs}}+\sum_{i=1}^{{\text{m}}_{n}}\left({\text{B}}_{ld}E_{ec}+B_{dt}E_{ec}\right)+\sum_{i=1}^{{\text{m}}_{n}+1}\left({\text{E}}_{dp}+E_{ap}\right)\;+B_{cd}\left(E_{ec}+E_{am}\;d_{j,R_{\max}}^{\beta }\right)+B_{pk}E_{ec}+B_{pk}\left(E_{ec}+E_{mp}\;d_{j,g}^{4}\right)+\sum_{i=1}^{m_{n,j}}\left(E_{ss}+E_{log}+\;{\text{E}}_{\text{cs}}+B_{ld}\left(2E_{ec}+E_{am}\;d_{i,j}^{\beta }\right)+B_{dt}(E_{ec}+E_{am}\;d_{i,j}^{\beta })\right)\right) \end{array}$$

Let B stand for $B_{ld},$ $B_{dt}$, $B_{pk}$ and β =2 for intra-cluster distance while β =4 for inter-cluster distance *i.e.*, clusterhead to BS:

(36) $$E_{net}=\left(\left(qE_{ss}+qE_{log}+\;{\text{qE}}_{\text{cs}}+2NBE_{ec}+N\left({\text{E}}_{dp}+E_{ap}\right)+Bq\left(3E_{ec}+\frac{2E_{am}}{3}\frac{L^{2}}{q}\right)\;+\text{Bq}E_{mp}\;d_{j,g}^{4}+N\left(E_{ss}+E_{log}+\;{\text{E}}_{\text{cs}}+B\left(3E_{ec}+\frac{4E_{am}}{3}\frac{L^{2}}{q}\right)\right)\right)\right)$$

Therefore, the optimal number of clusters $k_{opt}$ can be determined by setting derivative of $E_{net}$ in Equation (36) with respect to q to equal to zero $\frac{\partial E_{net}}{\partial q}=0$. After further derivation and simplification, the equation reduces to:

(37) $$q^{*}=\sqrt{\frac{4\text{N}E_{am}{\text{A}}_{N}}{3(E_{pp}+2E_{ec}+{\text{E}}_{tH})}}$$

where $E_{pp}=\left(E_{ss}+E_{log}+\;{\text{E}}_{\text{cs}}\right)/B$ denotes the energy costs per bit for sensing events, logging the readings data and sensing set of channels for detecting vacant channels, respectively.

### 3.5. Modelling of RL-Based Clustering

**Figure 5:**
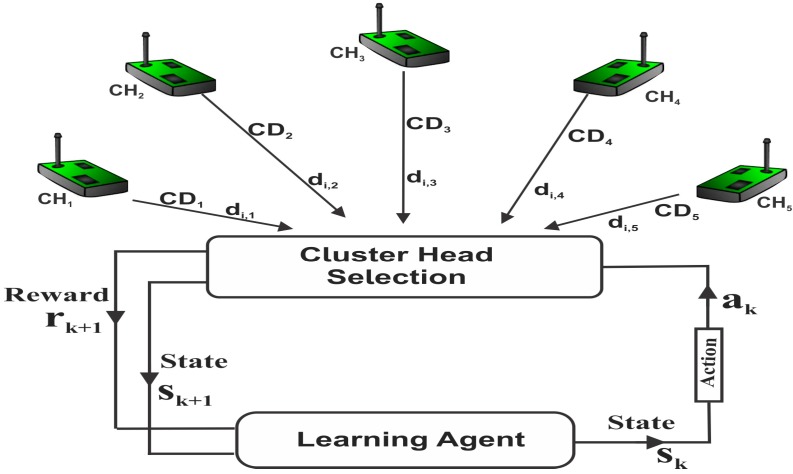
Model of reinforcement learning-based clustering.

The problem of selecting optimal clusterhead is formulated as a Markov Decision Process (MDP) where a SU learns the energy consumption and local decision accuracy for neighboring clusterheads and then selects an optimal cluster that minimizes energy consumption and improves sensing performance. A quadruple (S,T,A,ℛ) represents the Markov Decision Process (MDP) for selecting an optimal cluster in the network, S denotes set of states in the model of the operating environment $\text{s}=\left\{s_{1},s_{2},s_{3},s_{4}\ldots ,s_{n}\right\},\text{ s}\in S$, T denotes state transition function, A denotes a set of actions to be executed $a=\left\{a_{1},a_{2},a_{3}\ldots ,a_{n}\right\}$, a∈A, while ℛ denotes the state reward function r(s,a)∈ℛ [19]. Each of the SUs or the agent selects an action $a_{k}$ in every state $s_{k}$ of the model as shown in Figure 5. The selected action $a_{k}$ leads to sensing a set of channels, reporting local decisions to a clusterhead, computing the energy consumption and evaluating the local decision accuracy for the chosen cluster. Reward $r_{k+1}$ obtained from the computed energy consumption and local decision accuracy for state $s_{k}$ determines the next state $s_{k+1}$ and the next action $a_{k+1}$, k denotes the stage index of the process. The agent adopts an optimal policy π that maximizes the cumulative reward obtained from a known state experience and from exploitation of unknown states to select the optimal clusterhead.

**States:** The state $s_{k}$ of the MDP stands for the stage at which the agent selects a cluster among the neighboring clusterheads and determines the reward for taking an action $a_{k}$ in the state. The set |S| comprises of states equal to the number of neighbouring clusterheads plus an initialization state which initiates the state transition for selecting the clusterhead. Initialization of the clusterhead selection process begins at k=0, v=0 for $s_{\text{k}}^{t}=v$, where t= {0, 1, 2, ...,T} denotes the decision episode, k denotes the stage index and v∈ S denotes the current state number which indicates the selected clusterhead, if v≠0. Therefore, at every stage index k≠0 of the learning process, the state $s_{k}^{t}=\;v\in \;S$ for selecting an action $a_{k}^{t}\in \;A$ can be expressed as:

(38) $$s_{k}^{t}=v\;.\;I_{x}\left(s\right)\;$$

where $I_{x}\left(s\right)$ is an indicator function in that:

(39) $$I_{x}\left(s\right)=\{\begin{array}{c} 1,\;if\;a_{k}^{t}\neq 0\\ 0,\;otherwise \end{array}\;$$

For each episode t of the learning process, the agent i employs a softmax action selection strategy to select an action $a_{k}^{t}$, computes the state-action reward $r_{k}^{t}\in \;ℛ$ for the current state $s_{k}^{t}=v$, and then determines the subsequent state $s_{k+1}^{t}=h\in S$.

**Actions:** an action $a_{k}\;$in this context implies a strategic choice made by an agent for selecting a clusterhead $CH_{j}\;$among the neighbouring clusterheads $CH_{j}|j=1,\;2,\;3,\ldots ,n_{hg}$. The selected action $a_{k}^{t}=j\in A$ in every state $s_{k}^{t}=v\in \;S$ is expected to maximize the current reward $r_{k}^{t}$ for updating Q-value $Q_{k}^{t}\left(s_{k}^{t},a_{k}^{t}\right)$. If $Q_{k}^{t}=(s_{0}^{t}$,$\;a_{0}^{t},s_{1}^{t},a_{1}^{t},\ldots ,s_{k-1}^{t},a_{k-1}^{t},\;s_{k}^{t},a_{k}^{t})$ denotes the sequence of state-actions executed from $s_{0}^{t}\;$to $s_{k}^{t}$ in episodes t= {0, 1, 2, ..., T} and $CH_{k-1}^{t}$ denotes the corresponding set of selected clusterheads for the state-actions then the action taken can be formulated based on a stochastic process as $a_{k}^{t}=\sigma_{k}^{t}\left(Q_{k}^{t}\right)\in \left(A_{s_{k}^{t}}\right)$, where $A_{s_{k}^{t}}=A\backslash \{\left\{s_{k}^{t}\right\}\cup^{}CH_{k-1}^{t}$, denotes a set of selected actions, $\sigma_{k}^{t}$ denotes decision rule that maps the sequence of state-action $Q_{k}^{t}$ into a probability distribution $\Delta_{\sigma_{k}^{t}}\left(A_{s_{k}^{t}}\right)$ [3].

To achieve a policy π that maximizes long-term rewards, a Boltzmann distribution-based action selection strategy known as softmax is adopted to balance between exploration of random actions and exploitation of state-actions. The strategy selects an action that returns the highest estimated reward for the state-action values based on a probability p determined by a positive parameter $\tau^{t}\;$called temperature. The probability p can be expressed as [27]:

(40) $$p\left(s_{k}^{t},a_{k}^{t}=j\;\right)=\frac{e^{Q\left(s_{k}^{t},a_{k}^{t}=j\right)/\tau^{t}}}{\sum_{\bar{h}}^{\left|A_{s_{k}^{t}}\right|}e^{Q\left(s_{k}^{t},a_{k}^{t}=\bar{h}\right)/\tau^{t}}},\;j\in A_{s_{k}^{t}}\;$$

**Transition Probability**: The transition probability T: S × A × S….S →[0,1] maps the state-action transit to a probability of moving from current state $s_{k}^{t}$ to next state $s_{k+1}^{t}$ whenever an action $a_{k}^{t}$ is executed in the state $s_{k}^{t}$. Therefore, the transition probability $P_{m}\left(s_{k}^{t}/s_{k+1}^{t},\;a_{k}^{t}\right)\;$from current state $s_{k}^{t}=v$ to the next state $s_{k+1}^{t}=h\;$is a function of the action $a_{k}^{t}=j$ executed in the current state. However, the adopted learning algorithm which is Q-learning does not require transition probabilities.

**Reward function:** Reward $ℛ\;:S\;\times \;A\;\times S\ldots .\;S\;\to \;r_{k}^{t}$ is a key component of the MDP model that can be used to evaluate the state-action value for each episode and update the Q-table. It maps the state transition from state $s_{k}^{t}$ to subsequent state $s_{k+1}^{t}$ for the action $a_{k}^{t}\;$taken to an actual value reward [3]. In each episode, the agent selects an action $a_{\text{k}}^{t}=\text{j}$ to select clusterhead $CH_{j}$ in state $s_{\text{k}}^{t}=v$, computes its reward $r_{\text{k}}^{t}$ before moving to the next state $s_{\text{k}}^{t}=h$, and then restarts the state $s_{k}^{t}=0$ upon reaching the last state. The expected cumulative reward $r_{k+1}^{t}\;$can be computed based on agent’s reward for energy consumption $rw_{E,k+1}^{t}$ and reward for local decision accuracy $rw_{D,k+1}^{t}$ subject to the pairwise constraint condition $P_{w,k+1}^{t}$. Thus:

(41) $$r_{k+1}^{t}\;\left(s_{k}^{t},\;a_{k}^{t}\right)=\;\frac{1}{2}\left[\;rw_{E,k+1}^{t}+rw_{D,k+1}^{t}\;\right]I_{A}\left\{P_{w,k+1}^{t}\neq 0\right\}\;$$

where $k\;=0,\;1,\;2,\ldots ,k^{t}-1$ denotes the stage index at episode t, the indicator function $I_{A}\left\{x\right\}\;$can either be zero or one, depending on x. The pairwise constraint condition $P_{w,k+1}^{t}$ can be expressed as:

(42) $$P_{w,k+1}^{t}=\;\{\begin{array}{c} 1,\;M_{V,i}\cap H_{V,j}\neq \emptyset \\ 0,\;otherwise\; \end{array}$$

This means that the cumulative reward will be zero if the member node $MN_{i}$ and the chosen clusterhead $CH_{j}$ share no common vacant channel $M_{V,i}\cap H_{V,j}=\emptyset$. The reward for energy consumption $rw_{E,k+1}^{t}$ can be obtained from the member node’s $MN_{i}$ consumed energy ${\text{E}}_{tM}$ for transmitting $B_{dt}$bits of data packet to a neighboring clusterhead $CH_{j}$ and for performing cooperative channel sensing $E_{MN}^{cs}$. Thus, consumed energy is given as:

(43) $$E_{TC,j}\left(B_{dt},d\right)=\;E_{MN}^{cs}+\;{\text{E}}_{tM}\;=\overset{\;n_{z}}{\underset{m=1}{\sum }}\left(T_{cs}P_{ed}+{\text{E}}_{sp}\right)+B\left(3E_{ec}+2E_{am}\;d_{i,j}^{\beta }\right)\;$$

Therefore, a reward of one will be obtained for minimum energy consumption, a reward of half will be awarded for energy consumption less than the maximum consumed energy, while the reward for maximum energy consumed is zero. Thus, the reward for energy consumption is given as:

(44) rwE,k+1t={1, ETC,j=minETC,j∈ETCset(ETCset)12,  ETC,j<maxETC,j∈ETCset(ETCset)0, ETC,j=maxETC,j∈ETCset(ETCset)

State-action that leads to selection of a clusterhead that leads to low energy consumption receives a higher reward which translates into an increase in the corresponding Q-value and also its chances for likely selection in subsequent states. This means that less favorable clusters that lead to high energy consumptions are more likely to be excluded during the learning process.

Local decision on PU existence in a channel is usually prone to errors due to the channel’s propagation impairment which degrades the channel sensing performance. Therefore, the local decision accuracy determines the divergence of an individual member node’s sensing outcome compared to the cooperative sensing outcome. The reward for local decision accuracy can be obtained by comparing the local decision $LD_{i}^{z}\in LD_{i}$ made by member node $MN_{i}$ on PU existence on the channels with the cooperative decisions $CD_{j}^{z}\in CD_{j}$ which involves other member nodes MNs in the cluster $cl_{j}$. Therefore, when a set of local decisions $LD_{i}$ matches with a corresponding set of cooperative decisions $CD_{j}$, then a reward of one will be received, while local decisions $LD_{i}$ that agree with the majority of the corresponding cooperative decisions $CD_{j}$ receive half rewards, local decisions $LD_{i}$ that match the corresponding cooperative decisions $CD_{j}$ with less than half the number of the channels attract a reward of zero (*i.e*., do not earn a reward). Thus, the reward for local decision accuracy can be expressed as:

(45) rwD,k+1t={1, LDi∩CDj= nz 12, LDi∩CDj≥(nz/2)0, LDi∩CDj<(nz/2)

### 3.6. The EESA-RLC Algorithm

The algorithm begins with the clustering initialization phase which deals with clusterhead emergence as outlined in Table 1. All SUs (line 1 to line 20) sense a set of predefined channels (line 2), compute their clusterhead probabilities based on the number of vacant channels detected, percentage of clusterheads and their residual energy (line 3). If the clusterhead probability is greater or equal to the threshold, then a clusterhead announcement is broadcast (line 5) and the SU becomes a tentative clusterhead (line 6), otherwise, if the clusterhead probability is less than the threshold and it has heard the clusterhead announcement (line 7), then it waits for the final announcement (line 8) and the SU is a member node (line 9). Another set of SUs (line 10) perform iteration (line 11 to line 18). In each iteration, we increment the probability by a factor (line 12) and compare the result with the threshold (line 13). If it is clusterhead (line 15), it broadcasts an announcement (line 14). The Base Station selects an optimal number of clusterheads among the potential clusterheads (line 21) and broadcasts the list (line 24). The selected clusterheads broadcast advertisement packets to their neighboring SUs (line 24 to line 25).

**Table 1:** Initialization phase.

Algorithm 1: Cluster Head Emergence
Required: N, EnRe, q*, ψ, Enmx, ξ
1: **for** n←1 to N do
2: Sense Channels $\left(Ch_{z}|z=1,2,3,\ldots ,n_{z}\right)$
3: Compute pnch=EnRe φn ψ/Enmx nz
4: **if** $\left(p_{n}^{ch}\geq \psi \right)$ **then**
5: Broadcast $A_{pkt}$
6: CH=CH+1
7: **else if** $\left(p_{n}^{ch}<\psi \right)$ and Received $A_{pkt}$ **then**
8: Wait for final clusterhead announcement
9: MN=MN+1
10: **else**
11: **for** $l\leftarrow 1\;to\;N_{ir}\;do$
12: $p_{n}^{ch}\leftarrow p_{n}^{ch}+\;\xi \psi$
13: **if** $\left(p_{n}^{ch}\geq \psi \right)$ **then**
14: Broadcast $A_{pkt}$
15: CH=CH+1
16: Break
17: **end if**
18: **end for**
19: **end if**
20: **end for**
21: BS Selects $\;q^{*}$ from $CH_{n}|n=1,2,3,\ldots ,CH$
22: Broadcasts $H=\{CH_{n}|n=1,2,3,\ldots ,\;q^{*}\}$
23: **for** $\;j=1\;to\;q^{*}\;do$
24: Broadcast $A_{pkt}$
25: **end for**

The RL process shown in Table 2 begins immediately after the emergence of clusterheads by setting all state-action values Q(|S|,|A|) array and action to zero (line 2) and then carrying out a number of iterations (line 3 to line 30) up to maximum episodes $E_{ps}$ specified in the inputs (line 1). The state transition follows a sequential order (line 4) and is re-started upon reaching the number of clusters (line 28) *i.e.*, number of elements in $H_{n}$ (line 1). The softmax action selection strategy is employed (line 5) in each episode to select a clusterhead among the prospective clusterheads (line 7). This leads to sensing a set of channels $\left|S_{ch}\right|$ (line 8) and computing the energy consumptions (line 9) and then obtaining the associated rewards (line 11 to line 25). The state-action Q-values (line 26) are updated by the cumulative reward (line 10) and the final Q-matrix is determined after the last episode. The state-action that returns the maximum total cumulative reward value (line 31) denotes the optimal policy of selecting a clusterhead and the optimal cluster $CH_{j}^{*}$ is the index of the optimal policy which indicates the cluster (line 32).

**Table 2:** Reinforcement learning clustering.

Algorithm 2: RL Clustering
1: Input: $|S|,\;|A|,\;|S_{ch}|,\;E_{ps},\;|H_{n}|,\;n_{hg}$
2: *Initialize*: $Q(|S|,\;|A|)\leftarrow 0,\;k\leftarrow 0,\;a_{k}^{t}\leftarrow 0$
3: **for** $t\leftarrow 1\;to\;E_{ps}\;do$
4: k←k+1
5: $a_{k}^{t}\leftarrow \mathrm{actionStrategy}(s_{k}^{t},Q)$
6: **if** $a_{k}^{t}\neq 0\;\text{and}\;k\leq n_{hg}$ **then**
7: Select clusterhead $\left(CH_{j},a_{k}^{t}=j\right)$
8: Sense Channel $\left(|S_{ch}|\right)$
9: Compute $E_{TC}^{set}=\{E_{TC,j}|j=1,2,3,\ldots ,n_{hg}\}\;\text{for}\;|H_{n}|$
10: Compute reward $r_{k+1}^{t}\leftarrow \mathrm{Average}(rw_{E,k+1}^{t},rw_{D,k+1}^{t})$
11: **if** $E_{TC,j}=\min\;(E_{TC}^{set})$ **then**
12: $rw_{E,k+1}^{t}\leftarrow 1$
13: **else if** $E_{TC,j}<\max\;(E_{TC}^{set})$ **then**
14: $rw_{E,k+1}^{t}\leftarrow 0.5$
15: **else**
16: $rw_{E,k+1}^{t}\leftarrow 0$
17: **end if**
18: Compare decision $\left(L{\text{D}}_{i},CD_{j}\right)$
19: **if** $LD_{i}\cap CD_{j}=n_{z}$ **then**
20: $rw_{D,k+1}^{t}\leftarrow 1$
21: **else if** $LD_{i}\cap CD_{j}\geq (n_{z}/2)$ **then**
22: $rw_{D,k+1}^{t}\leftarrow 0$.
23: **else**
24: $rw_{D,k+1}^{t}\leftarrow 0$
25: **end if**
26: update $Q_{k}^{t}\leftarrow Q_{k}^{t}+\alpha [r_{k+1}^{t}+\gamma max_{a}(Q_{k+1}^{t})-Q_{k}^{t}]$
27: **else**
28: k=0
29: **end if**
30: **end for**
31: $[H_{opt,}I]\leftarrow \underset{s_{k}^{t}\in S,a_{k}^{t}\in A}{\max}\left|\sum_{t=1}^{E_{ps}}r_{k}^{t}\left(S_{k}^{t},a_{k}^{t}\right)\right|$
32: $CH_{j}^{*}\leftarrow |I|$

## 4. Performance Analysis and Evaluation

In this section, we analyze optimality of the EESA-RLC algorithm and evaluate its performance in terms of convergence, complexity and adaptability to model a free dynamic environment in achieving an optimal policy $\pi_{k}^{*}$ that minimizes energy consumption while enhancing vacant channel detection. The optimal policy $\pi_{k}^{*}$ maximizes the cumulative reward $r_{k}^{t}$ to achieve the optimal cluster $cl_{j}^{*}$ by choosing an optimal clusterhead $CH_{j}^{*}$ for the agent. 

In the simulation, we assume a moderate scale network consisting of 250 SUs uniformly distributed in a 90 m × 90 m square area and five PUs randomly deployed in the network. Each PU can operate on one channel such that SUs can only access unused licensed channels for data transmission. Each channel might be free or occupied at any time depending on PU activity. We also consider low power wireless sensor nodes in computing the energy dissipation for spectrum sensing, reporting to clusterheads and data transmission. All SUs are homogenous and uniformly distributed in the network area, therefore in our simulation and mathematical modeling, all radio parameter and energy dissipation values for processing the received signal samples, tuning the detector’s circuit to channel’s bandwidth and running the electronics circuit are the same. The parameter values used for both analysis and simulation as indicated in Table 3 are either computed based on parameter values obtained from the indicated references or originated from the sources indicated *in situ*.

The Q-learning and SARSA algorithms were implemented in MATLAB to evaluate the performance of the EESA-RLC algorithm for $E_{ps}=5000$ episodes [19]. We set the step size for the exploration of state-action pairs and for learning rate update to $\alpha^{k}=a/\left(b+k\right)$, while the discount factor is set to γ=0.9. The discount factor determines the level priority given to future rewards. A factor of zero makes an agent consider only immediate rewards [48]. 

**Figure 6:**
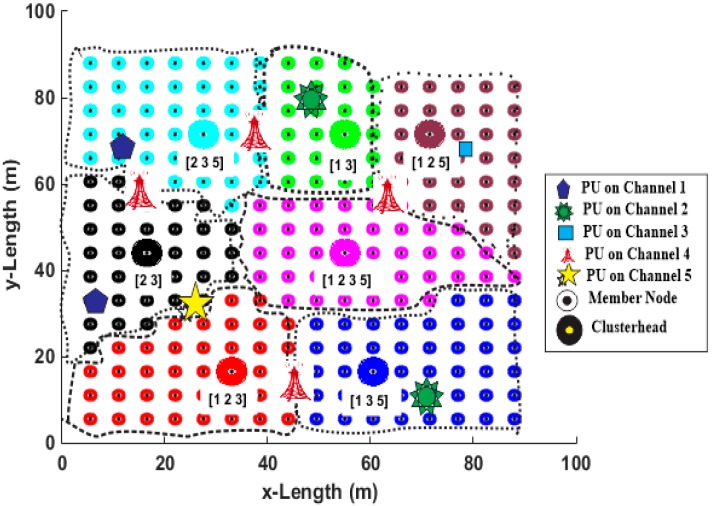
Pairwise spectrum-aware clustering result.

After extensive state-actions exploration and clusters exploitation during the learning phase, the EESA-RLC algorithm partitions the network into seven clusters $\left\{cl_{1},cl_{2},cl_{3},cl_{4},cl_{5},cl_{6},cl_{7}\right\}$ which translate into seven different clusterheads $\left\{CH_{1},CH_{2},CH_{3},CH_{4},CH_{5},CH_{6},CH_{7}\right\}$ as shown in Figure 6. The network consists of 250 uniformly distributed SUs and five PUs randomly deployed in the network.

The SUs form seven clusterheads and 243 member nodes. During the learning phase, each member node considers only clusterheads that are within its radio range instead of all seven clusters and chooses the optimal cluster among its neighborhood clusters. The seven clusters which are the optimal number for the network as determined through simulation as shown in Figure 6, are indicated by dashed circles with the corresponding common available channels inscribed in the circle. 

**Figure 7:**
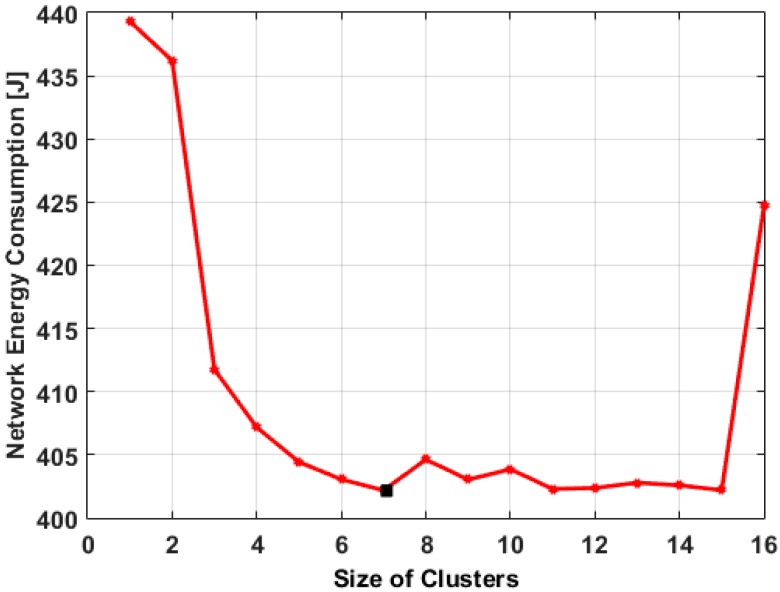
Energy consumption for clusters size.

To examine the effect of cluster size on the network energy consumption, we determined the energy consumed by both member nodes and their respective clusterheads for different cluster sizes through simulation, as shown in Figure 7. For a fixed number of nodes, when the number of clusters is too small (e.g., 3), each cluster would have a large number of member nodes that communicate with high transmission power. This increases the network energy consumption due to long intra-cluster distance communication between the member nodes and their clusterheads. 

**Table 3:** Value of parameters used in the simulation.

Parameter	Description	Value
N	Number of SUs cognitive radio sensor nodes	250
$n_{z}$	Number of licensed channels	5
ℳ	Number of primary users	5
BS	Base Station Coordinate	75,75
$d_{i,j}$	Maximum distances for intra-cluster transmission	≤45 m [45]
$d_{j,g}$	Maximum distance for inter-clusters transmission	≤150 m [45]
$E_{psd}$	Number of episodes	5000
$\alpha^{k}$	Step Size for learning rate	a/(b+k), a = 1, b = 10;
γ	Discounted factor	0.9
pkt	Packet Size	32 byte
${\text{E}}_{n}^{\text{mx}}$	Initial Energy	1250 mJ
$B_{W}$	Channel Bandwidth	$Ch_{1}:650$ MHz $Ch_{2}:600$ MHz $Ch_{3}:200$ MHz $Ch_{4}:750$ MHz $Ch_{5}:50$ MHz
ε	Tradeoff between exploration and exploitation	0.7
$E_{sp}$	Energy dissipation: signal samples processing	150 nJ/bit [49]
$E_{ap}$	Energy dissipation: data aggregation	5 nJ/bit [45]
$E_{ec}$	Electronics dissipation: electronics circuit	50 nJ/bit [50]
$E_{ss}$	Energy dissipation: event sensing	33.75 μJ/bit [45]
$E_{log}$	Energy dissipation: data logging	81.4 μJ/bit[45]
$E_{am}$	Energy dissipation: amplifier, intra-cluster	77 pJ/bit/m^2^ [45]
$E_{mp}$	Energy dissipation: amplifier, inter-cluster	0.002 pJ/bit/m^4^ [45]
$P_{ed}$	Power consumption: tuning detector’s circuit	40 mW [49]
$E_{dp}$	Energy dissipation: data fusion	0.187 μJ/bit [49]
η	Power amplifier drain efficiency	92.4%[51]

On the other hand, for large number of clusters (e.g., 15), the network energy consumption is relatively small, but inter-cluster communication consumes relatively high energy because of the excessive number of clusterheads which in reality consume much more energy than member nodes. Therefore, network energy consumption can be minimized by determining the optimal number of clusters that balances energy consumption for inter-cluster and intra-cluster communications. The result shows that at the minimum network energy consumption, the optimal number of clusters is seven.

### 4.1. Optimality of EESA-RLC Algorithm

The EESA-RLC algorithm allows SUs to learn and adapt to the dynamic environment to achieve an optimal solution through an optimal policy. The optimal solution means the optimal clusterhead $CH_{j}^{*}\in H_{n}$ selected by the cluster member node $MN_{i}\;$through the optimal policy $\pi^{*}\;$that maximizes cumulative reward $r_{k}^{t}$. The necessary conditions required to achieve the optimal solution is presented in the following proposition and proof.

**Proposition 1.** *Consider a given set of neighboring clusterheads* $H_{n}=\left\{CH_{j}|j=1,\;2,\;3,\ldots \ldots n_{hg}\right\}$ *located at different distances* $dis_{n}=\left\{d_{ij}|j=1,\;2,\;3,\ldots ,n_{hg}\right\}$ *from a member node* $MN_{i}$*. The corresponding set of energy consumptions* $E_{TC}^{set}\left(B_{dt},d\right)=\{E_{TC,j}|j=1,\;2,\;3,\ldots ,n_{hg}\}$ *for the member node* $MN_{i}$ *to transmit its reading data to each of the clusterhead* $CH_{j}|j=1,2,3\ldots .n_{hg}$ *is a function of distance* $d_{ij}$ *to the clusterhead. Each clusterhead*$\;CH_{j}$ *and member node* $MN_{i}$ *sense set of channels* $S_{ch}=\left\{Ch_{z}|z=1,\;2,\;3,\ldots \ldots n_{z}\right\}$ *and detect* $M_{V,i}=\left\{Ch_{z}|z=1,\;2,\;3,\ldots \ldots n_{mv}\right\}$ *and* $H_{V,j}=\left\{Ch_{z}|z=1,\;2,\;3,\ldots \ldots n_{hv}\right\}$ *set of vacant channels respectively. If a selected clusterhead*$\;CH_{j}$ *with* $\underset{d_{ij}\in dis_{n}}{\min}d_{ij}$ *and*$\;\underset{M_{V,i},H_{V,j}\epsilon S_{ch}}{\max}\left(M_{V,i}\cap H_{V,j}\right)\;$ *maximizes cumulative reward* $R_{k}=\frac{1}{2}\left[\;rw_{E,k+1}^{t}+rw_{D,k+1}^{t}\;\right]$ *such that* $R_{k}=\underset{s_{k}^{t}\in S,\;a_{k}^{t}\in A}{\max}\left|\overset{E_{ps}}{\underset{t=1}{\sum }}r_{k}^{t}\;\left(s_{k}^{t},\;a_{k}^{t}\right)\right|$*, then there exist an optimal action selection* ${\pi^{*}}_{k}=\left(a_{1}^{*},\;a_{2}^{*},\ldots \ldots .a_{k}^{*}\right)$ *that selects optimal clusterhead* $CH_{j}^{*}$, where $a_{k}^{*}=\underset{a_{k}\in A}{argmax}\;r_{k}\left(s_{k},\;a_{k}\right)$.

**Proof.** In every state $s_{k}$, a member node $MN_{i}$ selects a clusterhead $CH_{j}\in H_{n}$ among the set of clusterheads to determine a set of common vacant channels $M_{V,i}\cap H_{V,j}$ and energy consumption $E_{TC,j}\left(B_{dt},d\right)\in E_{TC}^{set}\left(B_{dt},d\right)$ for transmitting data to the clusterhead over distance $d_{ij}$ through the vacant channels. The selected clusterhead $CH_{j}$ with minimum distance $\underset{d_{ij}\in dis_{n}}{\min}d_{ij}$ must minimize energy consumption $E_{TC,j}\in E_{TC}^{set}\left(B_{dt},d\right)$, maximize vacant channels detection $\underset{M_{V,i},H_{V,j}\epsilon S_{ch}}{\max}\left(M_{V,i}\cap H_{V,j}\right)\;$ and satisfy the pairwise constraint condition $P_{w,k+1}^{t}$ such that $M_{V,i}\cap H_{V,j}\neq \emptyset$ and obtain the highest reward value for the energy consumption reward $rw_{E,k+1}^{t}$ and highest reward value for the local decision accuracy reward $rw_{D,k+1}^{t}$ to achieve the maximum cumulative reward value $R_{k}=\underset{s_{k}^{t}\in S,\;a_{k}^{t}\in A}{\max}\left|\overset{E_{ps}}{\underset{t=1}{\sum }}r_{k}^{t}\;\left(s_{k}^{t},\;a_{k}^{t}\right)\right|$. If j denotes the index of the selected clusterhead $CH_{j}\in H_{n}$ in states $s_{1}to\;s_{k}$ that maximizes the cumulative reward $R_{k}$, then for each state $s_{k},\;\text{j}=\underset{a{\;}_{k}\in A}{argmax}\;r_{k}\left(s_{k},a_{k}\right)$ which denotes the optimal action selection policy $\pi^{*}=a_{k}^{*}\;$and the optimal clusterhead $CH_{j}^{*}\in H_{n}$.

### 4.2. Convergence and Computational Complexity of the EESA-RLC Algorithm

**Figure 8:**
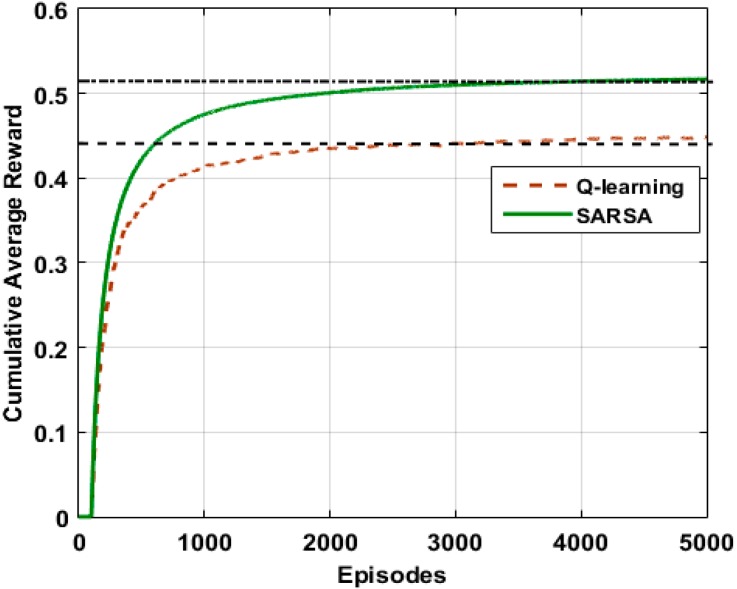
Expected cumulative rewards and algorithms convergence.

The algorithm converges to an optimal solution after adequate exploration and exploitation of state-actions pairs. The algorithm’s convergence period is a function of some parameter values which include action selection strategy, discount factor and learning rate. We simulate the Q-learning and SARSA RL algorithms to examine the convergence of the algorithms over $E_{psd}=5000$ episodes and evaluate their performance as shown in Figure 8. The result indicates that both the learning algorithms converged to the optimal solution but after different numbers of episodes. The SARSA learning algorithm converged to the optimal value after $E_{psd}=3020$ episodes and achieved a maximum average expected cumulative reward value of $r_{Av}^{t}=0.52$ which is much higher than that of the Q-learning algorithm. On the other hand, the Q-learning algorithm converged to an optimal solution at $E_{psd}=2020$ which is much lower than SARSA and achieved a maximum expected cumulative reward value of $r_{Av}^{t}=0.44$. This suggests that the Q-learning algorithm converges to optimal solution in a relatively short learning period because of its reliance on an action selection strategy rather than cluster exploration to update its estimated optimal policy. In contrast, cluster explorations while updating the Q-value slows the convergence of the SARSA algorithm due to the extension of its learning period, but this of course yields a better expected accumulative reward. It can be concluded that learning period has a significant effect on the convergence of the algorithms, increasing as the number of episodes increases which in turn decreases the learning rate and therefore, the algorithm converges slower.

**Figure 9:**
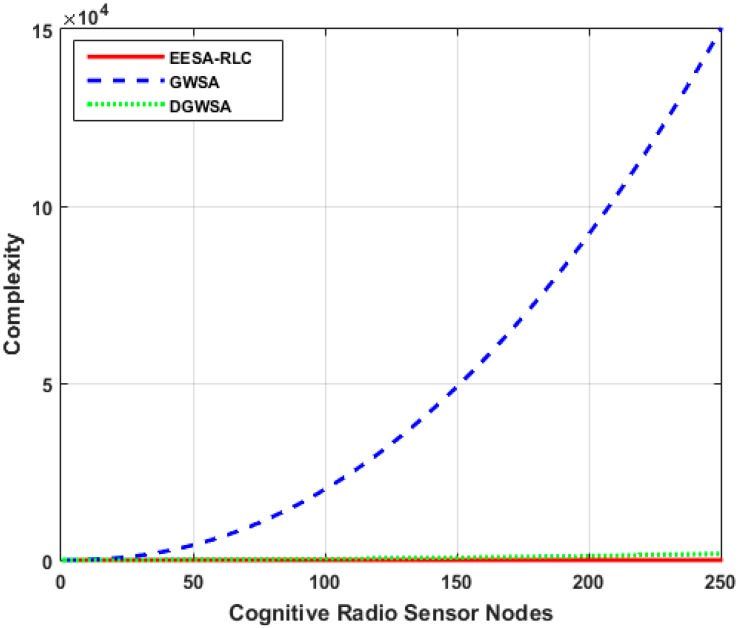
Comparison of computational complexity between EESA-RLC and other approaches.

Optimal clustering can be achieved through many techniques. While some techniques employ computationally infeasible approaches such as exhaustive search techniques which try all possible options for efficient clustering and select the best option among them, while others employ less computationally complex approaches based on heuristic techniques such as hierarchical, k-means, c-means and fuzzy clustering, *etc.* For example, the GWSA approach performs a high number of iterations to merge the nearest clusters in the nodes’ proximity matrix till an optimal solution is achieved [17]. However, its overall computational complexity $O\left(N^{2}\;logN\right)$ is extremely high and increases proportionally with increase in size of the number of cognitive radio sensor nodes which make it impractical to implement in a large scale network. Distributed Group Wise Spectrum Aware (DGWSA) offers relatively low complexity as shown in Figure 9 [18]. This approach merges multiple neighboring cluster pairs at every iteration instead of using a proximity matrix and its complexity largely depends on the size of neighboring clusters instead of network size. Therefore, we compare the computational complexity of our algorithm with that of the GWSA and DGWSA algorithms by increasing the size of the cognitive radio sensor nodes as shown in Figure 9. The result shows that the GWSA algorithm offers high computational complexity, which increases with size of the network, while our algorithm and the DGWSA one achieve much lower complexities that are largely influenced by the number of neighboring clusters.

**Figure 10:**
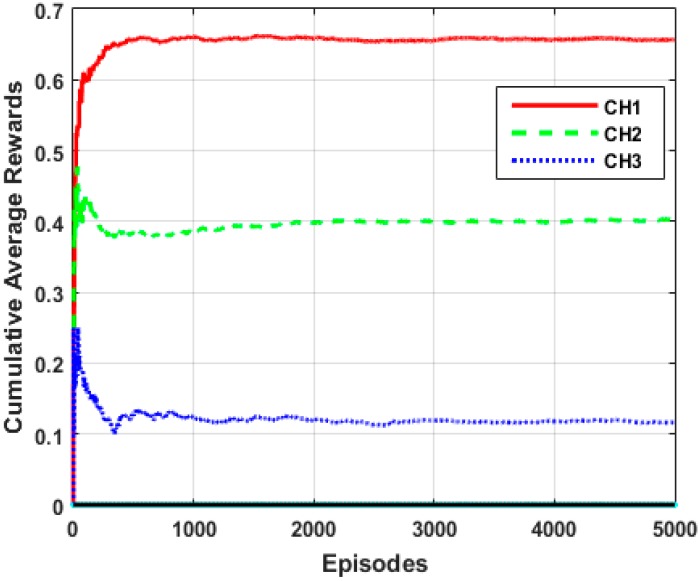
Average cumulative rewards for clusters.

**Figure 11:**
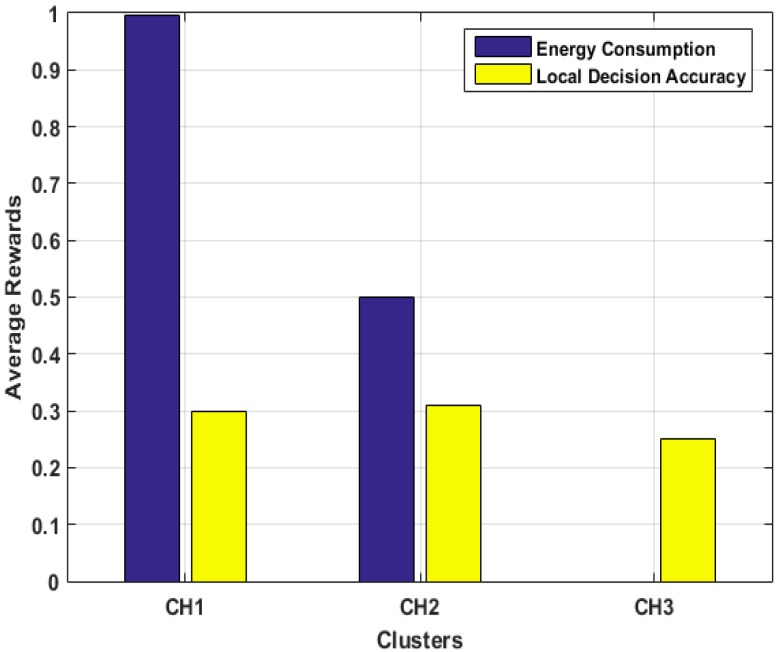
Average rewards for energy consumption and local decision.

We employed Q-learning to evaluate the performance of our algorithm in terms of learning and adaptability to a dynamic environment in achieving an optimal solution. Our choice for Q-learning is due to its faster convergece which requires a shorter learning period. We examined the performance of a single MN that learns and determines cumulative average rewards for each selected clusterhead in a total number episodes of $E_{psd}=5000$, as shown in Figure 10. We also examined the influence of the two metric functions, *viz* energy consumption and local decision accuracy, on the optimal cluster selection as shown in Figure 11. 

The cumulative average reward represents the average reward obtained for energy consumption and local decision accuracy during the learning process. Figure 10 shows cumulative average for the three clusterheads $CH_{1},CH_{2},CH_{3}$ that are within the radio range of the MN without the remaining four clusterheads $CH_{4},CH_{5},CH_{6},CH_{7}$ that have no direct link with the MN. The result indicates that clusterhead $CH_{1}$ attracts the highest cumulative average reward of $r_{Av}^{t}=0.65$, followed by clusterhead $CH_{2}$ which attracts cumulative average reward of $r_{Av}^{t}=0.4$, while clusterhead $CH_{3}$ receives the lowest cumulative average reward of $r_{Av}^{t}=0.12$. This indicates that the agent learns and adapts to the environment through exploration of the neighbouring clusterheads and exploitation of actions that attract favourable rewards to return clusterhead $CH_{1}$ as the optimal clusterhead.

**Figure 12:**
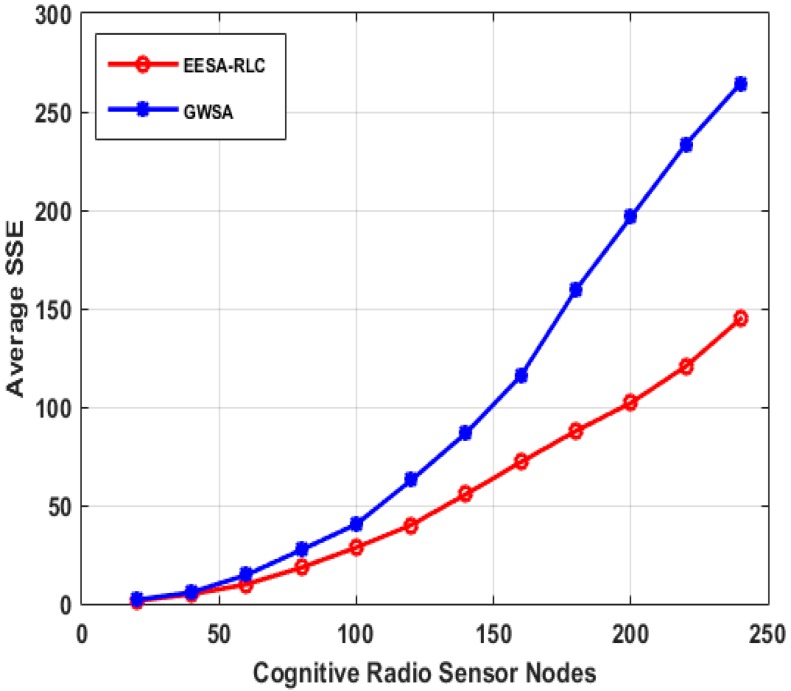
Average SSE for CRSN size.

**Figure 13:**
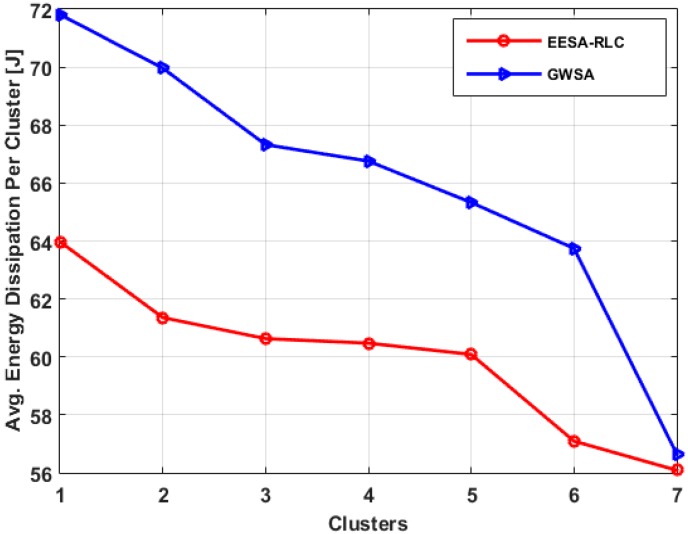
Average energy consumption for clusters.

**Figure 14:**
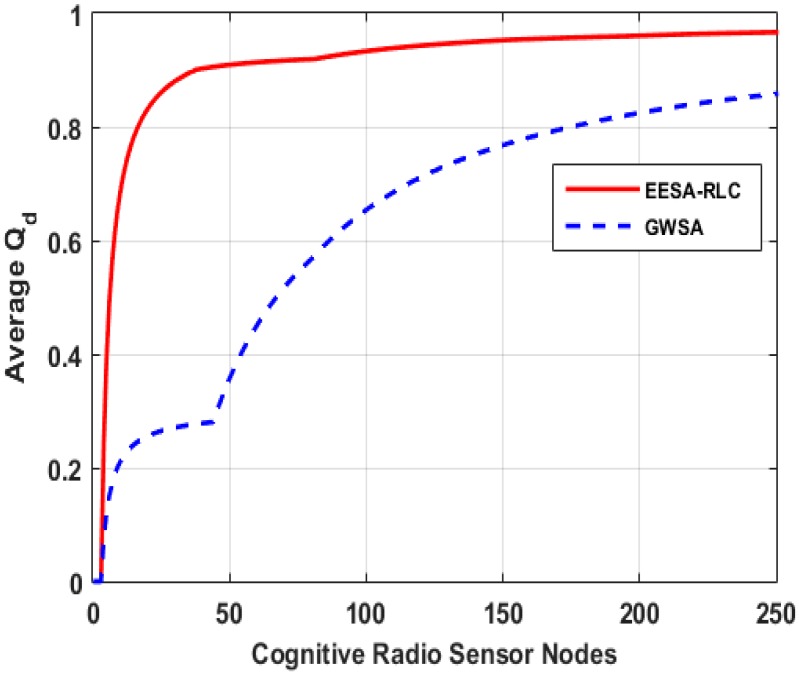
Average cooperative probability of detection for various CRSN size.

Figure 11 shows the effect of energy dissipation and local decision accuracy on selecting a clusterhead during the learning phase and choosing the optimal clusterhead. The agent learns the energy and cooperative sensing costs for each of the neighboring clusterhead and then selects the most favorable clusterhead that satisfies the pairwise constraints and minimum energy dissipation requirements as the optimal clusterhead. The local decision accuracy indicates the success of individual MN’s local decision about channels occupancy in respect to cooperative decision. In this context, local decisions are considered to be accurate when local decisions about a set of channels agree with the cooperative decisions irrespective whether the channels are available or not. Therefore, it can be deduced from the result, all the three clusterheads $CH_{1}$,$\;CH_{2}$ and$\;CH_{3}$ have satisfied the pairwise constraint which requires at least one common available channel between the MN and the clusterhead but only clusterhead $CH_{1}$ satisfied the minimum energy consumption requirement with the highest reward value $rw_{E}^{t}=0.98$. This suggests that significant amount of energy can be saved when the MN selects $CH_{1}$ as the optimal cluster as compared to $CH_{3}$ which attracts zero reward $rw_{E}^{t}=0$ for energy consumption. Therefore, it is extremely important to consider not only cooperative sensing success, but also energy consumption when choosing the optimal clusters. To evaluate the performance of our algorithm, we used GWSA [17] clustering as the benchmark for comparison, since it also considers spectrum-aware constraints in the network clustering and converges to optimal clusters. We first implemented the algorithms and obtained the optimal clustering through simulation, and then compared the performance of GWSA with our algorithm in terms of network energy minimization and spectrum sensing enhancement. Based on member nodes’ distances to their respective clusterheads in each cluster and clusterheads’ distances to the BS obtained from each of the clustering scheme, we determined Sum of Square Error (SSE) for the network, and computed network energy consumption and determined probability of detection for the two schemes as shown in Figure 12, Figure 13 and Figure 14 respectively.

Figure 12 compares the average SSE for different numbers of member nodes for the two clustering schemes. The SSE is a key component for determining the performance of clustering schemes in terms of network energy efficiency. Less SSE translates into minimum network energy consumption, which means more energy efficiency can be achieved with the clustering schemes that has less SSE. The result shows that SSE increases along with increase in number of member nodes. It is observed that our clustering scheme achieves much lower SSE than the GWSA. For example SSE for 200 member nodes is approximately 100 which is 50% less than that of GWSA for the same size of member nodes. This suggests that our approach is more energy efficient. 

Figure 13 further reveals the performance improvement of our approach over the GWSA in terms energy efficiency. The result indicates that average energy dissipation decreases with increase in size of clusters. It is evident from the result that our approach achieved the least average energy dissipation compared with the GWSA approach. For example, the average energy dissipation for a five-cluster size of our approach is about 60 J, which is 8.4% lower than that of GWSA approach for the same cluster size. Furthermore, the total network energy dissipation based on our clustering scheme is 417.3 J, while that of GWSA is 458.8 J. This implies that an energy savings of 9% can be achieved with our approach. This shows that the RL-based approach learns energy dissipation for each neighboring cluster through exploration of the clusters and chooses the most favorable cluster that attracts low dissipation through exploitation. 

Figure 14 compares the average probability of detection Qd for the two clustering schemes with different numbers of cognitive radio sensor nodes. The result indicates that our approach performs much better than the GWSA clustering scheme in terms PU detection. This is because the GWSA approach does not incorporate cooperative spectrum sensing which is vital for improving PU detection. It is observed that the average cooperative probability of detection Qd of our approach rapidly reaches a relatively high value that satisfies the required detection accuracy of ${\bar{Q}}_{d}\geq 0.9$ at the initial stage and then increases slowly with increasing number of cognitive radio sensor nodes. This suggests that multi-user sensing diversity exploration is crucial for enhancing PU detection and discovering of more spectrum opportunity.

## 5. Conclusions

In this paper, we propose a novel spectrum-aware clustering algorithm based on reinforcement learning to minimize network energy consumption and enhance channel sensing in cognitive radio sensor networks. We first modelled the network energy consumptions in terms of cooperative channel sensing, and inter-cluster and intra-cluster data communication energy consumptions, and then show that network energy consumptions can be minimized by determining an optimal number of clusters that balances energy consumption for inter-cluster and intra-cluster communications. The problem of nodes to choose their respective optimal clusters is formulated as a Markov decision problem and the results obtained show that the algorithm is capable of adapting to a dynamic environment and converging to an optimal solution.

We also showed that pairwise constraints can be implemented in spectrum-aware clustering to improve primary user detection. Also the energy cost and local decision accuracy have a significant influence on determining the optimal cluster. We further showed through simulations the performance improvement of our approach over groupwise constraint-based algorithms in terms of energy efficiency, channels sensing performance and computational complexity, which are vital to resource constrained devices such as CR-WSN. 
